# Combination of Active Learning and Semi-Supervised Learning under a Self-Training Scheme

**DOI:** 10.3390/e21100988

**Published:** 2019-10-10

**Authors:** Nikos Fazakis, Vasileios G. Kanas, Christos K. Aridas, Stamatis Karlos, Sotiris Kotsiantis

**Affiliations:** 1 Wired Communications Lab, Department of Electrical and Computer Engineering, University of Patras, 26504 Achaia, Greece; vaskanas@upatras.gr; 2 Educational Software Development Lab, Department of Mathematics, University of Patras, 26504 Achaia, Greece; char@upatras.gr (C.K.A.); stkarlos@upatras.gr (S.K.); sotos@math.upatras.gr (S.K.)

**Keywords:** active learning, semi-supervised learning, self-training, classification, combination of learning methods

## Abstract

One of the major aspects affecting the performance of the classification algorithms is the amount of labeled data which is available during the training phase. It is widely accepted that the labeling procedure of vast amounts of data is both expensive and time-consuming since it requires the employment of human expertise. For a wide variety of scientific fields, unlabeled examples are easy to collect but hard to handle in a useful manner, thus improving the contained information for a subject dataset. In this context, a variety of learning methods have been studied in the literature aiming to efficiently utilize the vast amounts of unlabeled data during the learning process. The most common approaches tackle problems of this kind by individually applying active learning or semi-supervised learning methods. In this work, a combination of active learning and semi-supervised learning methods is proposed, under a common self-training scheme, in order to efficiently utilize the available unlabeled data. The effective and robust metrics of the entropy and the distribution of probabilities of the unlabeled set, to select the most sufficient unlabeled examples for the augmentation of the initial labeled set, are used. The superiority of the proposed scheme is validated by comparing it against the base approaches of supervised, semi-supervised, and active learning in the wide range of fifty-five benchmark datasets.

## 1. Introduction

The most common approach established in machine learning (ML) is supervised learning (SL). Under the SL schemes, classifiers are trained using purely labeled data. In contrast with the problem complexity, the performance of such schemes is directly analogous to the amount and the quality of labeled data which are used at the training phase. In a large variety of scientific domains, such as object detection [1], speech recognition [2], web page categorization [3], and computer-aided medical diagnosis [4,5,6] vast pools of unlabeled data are often available. Though, in most cases labeling data can be costly and time-consuming, as human effort and expertise are required to annotate the available data. Many research works [7] exist focusing on techniques with the aim of exploiting the available unlabeled data especially in favor of classification problems. The most common learning methods incorporating such techniques are active learning (AL) and semi-supervised learning (SSL) [8]. Both AL and SSL share an iterative learning nature, making them a perfect fit for constructing more complex combination learning schemes.

**Figure 1:**
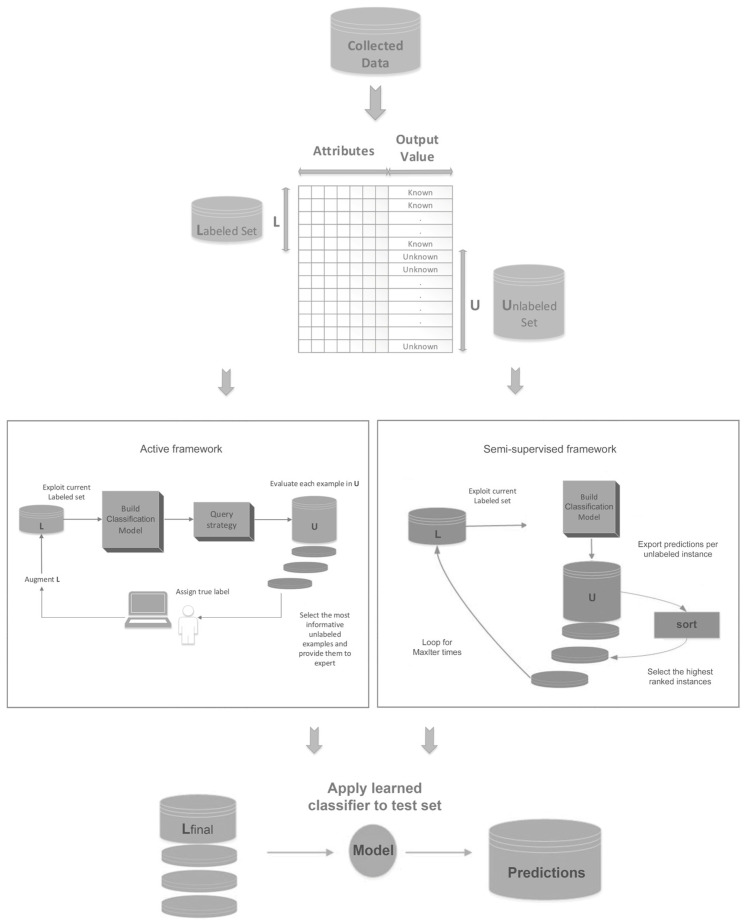
The general frameworks of active learning and semi-supervised learning along with their shared elements.

The primary goal of this paper is to put forward a new AL and SSL combination algorithm in order to efficiently exploit the plethora of available unlabeled data found in most of the ML datasets and provide an improved classification framework. The general flow of AL and SSL frameworks is presented in Figure 1. Both methods utilize an initial pool of labeled and unlabeled examples with the goal of efficiently augmenting the available knowledge. AL and SSL frameworks, in most cases, operate under an iterative logic aiming to predict the label in the most appropriate unlabeled examples. While the former method annotates the unlabeled instances by interactively querying a human expert based on a variety of querying strategies, the latter attempts to automatically produce the labels of unlabeled examples by exploiting the previously learned knowledge and a wide range of unlabeled instances selection criteria. After the successful augmentation of the initial labeled set, a final model is constructed in both cases with a view to the application on the unknown test cases.

As both methods share a lot of key characteristics, a major effort is now needed to combine the two learning approaches. The main contribution of the proposed algorithm is the employment of a self-training scheme for the combination of AL and SSL utilizing the fast and effective metrics of the entropy and the distribution of the prediction probabilities of the available unlabeled data. The plethora of experiments carried out, also play a major role in the validation of the proposed algorithm. The proposed method is examined through a number of different individual base learners, where the ensemble learning technique is also explored as the aggregated models tend to produce more accurate predictions and are commonly used in today’s applications [9,10].

**Figure 2:**
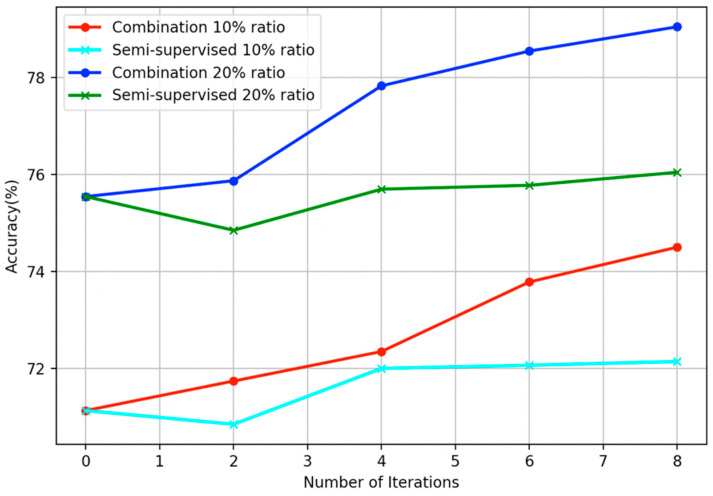
Progression of accuracies in relation to the number of iterations executed for the proposed combination scheme and its semi-supervised counterpart, utilizing support vector machines (SVMs) as base learner, applied on the Spambase dataset using two different labeled ratios.

Real-world case scenarios where AL and SSL combination methods can be applied include natural language processing (NLP) problems to which a lot of labeled examples are required to effectively train a model and also vast amounts of unlabeled data can be mined. Common applications on the NLP field are part of speech tagging, named entity recognition, sentiment analysis [11], fraud detection, and spam filtering. Especially, a number of AL [12], SSL, and combinations [13] of them have been proposed in the spam filtering domain. In Figure 2, an application on the Spambase [14] benchmark dataset briefly presents the accuracy improvement for the proposed scheme as the algorithm’s iterations progress. With regard to the base algorithm learner, the support vector machines (SVMs) [15] classifier was embedded. For comparison, in the same figure, the corresponding SSL part of the algorithm was fed with the same amount of unlabeled data to obtain only the semi-supervised accuracy.

The rest of this research work is organized as follows: In Section 2, the related work on similar classification methods is discussed. Following in Section 3, the proposed method is presented along with the exact algorithm implemented. An attempt to evaluate the efficacy of the combination scheme is made in Section 4, where extensive experimentation results can be found. Moreover, in this section, the average accuracies of the classifiers applied on the combination scheme are also briefly compared. In Section 5, a modification of the scheme is explored. The research conclusions are conferred in Section 6, where a number of areas to be explored as future work are mentioned. Finally, a software implementation of the wrapper algorithm is found in the Appendix A through the accompanying link.

## 2. Related Work 

AL can be considered one of the most promising approaches for improving the performance of a prediction model in real-world scenarios where large amount of data exists, but their labeling is costly or infeasible [7]. AL assumes that human experts will be available to provide ground-truth labels for the unlabeled instances. Therefore, the philosophy of AL is to minimize the number of queries with the explicit goal to focus the labeling effort in the most profitable or informative instances, in other words, to minimize the training cost of the model [16]. Finally, these manually annotated samples are merged with the training dataset to get the highest classification accuracy. Several query strategies [7,17] have been proposed to measure the informativeness or the representativeness of the data. Informativeness-based strategies measure the contribution of an unlabeled instance on the uncertainty reduction of a statistical model, while representativeness-based strategies measure the instance contribution on representing the underlying structure of input patterns. The most commonly used query strategies can be considered certainty-based sampling, query-by-committee, and expected error reduction. In the first type of strategy, a single model is trained and the human expert (annotator) is queried to label the least confidence unlabeled instances based on the pre-trained model. The query-by-committee strategy involves more than one active learner (classification models) to be trained for the classification task. The unlabeled instances about which these models disagree the most are selected for human annotation. The third strategy is a decision-theoretic approach aiming to estimate the potential of the model’s generalization error reduction. In other words, a model is trained and used to estimate the expected future error of the unlabeled samples. Then, the instances with the minimal future error (risk) are selected and delivered for manual labeling. The effectiveness of AL and various query strategies has been shown in typical classification tasks, such as text classification [18], speech recognition [19], speech emotion classification [20], audio retrieval [21] to name a few.

In contrast to AL, SSL aims to automatically exploit unlabeled data in addition to labeled data to improve learning performance, without human intervention. In SSL, two basic assumptions about the data distribution are considered. The first assumes that data are inherently clustered, meaning that instances belonging to the same cluster have the same label. The other one assumes that data lie on a manifold, meaning that nearby samples have similar predictions. The idea behind both is that similar data points should have similar outputs and the unlabeled instances can expose similarities between these data points. Many different SSL methods have been designed in machine learning, including mainly transductive support vector machines [22], graph-based methods [23,24,25], co-training [26], self-training [1]. In the self-training scheme, the classification model is used to predict the labels of a portion of the unlabeled instances and, consequently, the most confident ones are added to the initial training dataset repeatedly until convergence. Rather than just relying on a unique model, in co-training [26] ensemble method is employed. For each model, separate feature sets (or views) of the same labeled data are used for training. Then, like self-training, the most confident predictions of each classifier on the unlabeled data are used to iteratively construct additional labeled training data. The co-training paradigm relies on three assumptions about the views, i.e., sufficiency, compatibility, and conditional independence [26]. On the other hand, graph-based methods treat all the samples (both labeled and unlabeled) as connected vertices (nodes) in a graph, aiming to connect these nodes, in other words, to weight these node-to-node pairwise edges by similarities between the corresponding sample pairs. Finally, minimum energy optimization is used to propagate labeling from the labeled to the unlabeled nodes.

Although AL has led to the reduction of the human labeling burden, without sacrificing the model’s performance [7], it is still inefficient in some situations, e.g., the acquisition of a large amount of human annotations is impractical or not feasible at all. Thus, SSL comes in handy by minimizing the unlabeled data that will be fed to the human annotator. Specifically, human experts are required to label only those instances with the lowest certainty (as determined by the AL algorithm), while the remaining instances are automatically labeled by a machine annotator (by the SSL algorithm). Indeed, several studies have been proposed that combine AL and SSL under the same methodology. One of the first attempts [27] was in the text classification field, where expectation maximization was employed along with pool-based active learning. Later, Muslea et al. proposed the combination of co-testing and co-training showing improved classification accuracy in Web pages and pictures classification. During co-training, two classifiers are trained separately on two different views, and only the contention points, i.e., the unlabeled instances in which the classifier disagrees the most, were selected for human annotation. Finally, expectation maximization co-training (co-EM) was employed to automatically label instances that showed a low disagreement between the two classifiers. Other studies exploited certainty-based AL with self-training aiming to manual labeling with minimum human cost in spoken language understanding [28], natural language processing [29], sound classification [30], disease classification [31] and cell segmentation [32]. In another study [33], the authors addressed the problem of imbalanced training data in object detection. First, a simple object detection model was trained using a small portion of perfect samples instead of using the entire training dataset, while the imperfect samples were partitioned into several batches. Then, a batch-mode learning of AL and SSL combination was employed by integrating the uncertainty and diversity criteria from the concept of AL and the confidence criterion from that of SSL.

## 3. Proposed Method

The proposed method constitutes a combination of AL and SSL approaches, in order to leverage the advantages of both techniques. A mixed self-training method is employed utilizing the entropy of unlabeled instances, with the aim to identify the most confusing instances in the case of active round, while in the semi-supervised round the internal learner’s distribution of probabilities for all possible labels per each instance is exploited as a sorting mechanism for the selection of the most confident examples.

**Algorithm 1:** Combination Scheme
1:LOAD the dataset ***D*** and construct the labeled set ***L*** and the unlabeled set ***U***2:INITIALIZE the classifier ***CLS***3:CALCULATE the labeled ratio ***R = size(L)/size(L+U)***4:DEFINE the maximum number of iterations ***MaxIter***5:DEFINE the maximum percentage of unlabeled examples to be added in each iteration ***T*** in respect with ***R***6:SET ***maxUnlabPerIter*** = ***T*** * ***R*** * size(***D***)7: 8:SET ***i*** = 09:WHILE ***i***<***MaxIter*** *AND size(****U*^***i***^)**>0: /* where **U^0^**= **U** */10: Train(***CLS)*** on the current labeled set ***L^i^*** /* where ***L^0^***=***L*** */11: IF ***i*** modulo 2 == 0:12:  Classify(***U^i^***) using ***CLS*** and construct matrix ***M_pr_*** containing corresponding prediction probabilities along with the predicted labels13:  SORT ***M_pr_*** descending according to the prediction probabilities14:  STORE the top ***maxUnlabPerIter*** instances of ***M_pr_*** in a matrix ***M_final_***15:   /* now containing the most confident instances along with their predictions */16: ELSE:17:  Calculate the distribution_of_probabilities(***U^i^***) and return a matrix ***DistU^i^***18:  Calculate the entropy(***DistU^i^***) for each element and return a matrix ***EntrU^i^***19:  SORT ***EntrU^i^*** descending according to their entropies20:  Label the top ***maxUnlabPerIter*** using human expertise21:  STORE the top ***maxUnlabPerIter*** instances along with their labels in a matrix ***M_final_***22:   /* now containing the most confusing instances along with their true labels */23: END_IF24: Augment(***L^i^***) by adding ***M_final_*** instances25: Clean(***U^i^***) by removing ***M_final_*** instances26: SET ***i*** = ***i*** + 127:END_WHILE28: 29:Train(***CLS***) using ***L^augmented^* (☰ *L^last iteration^*)**30:LOAD the unknown test cases as ***Test_set_***31:Classify(***Test_set_***) using ***CLS*** to produce the final predictions


Uncertainty-based metrics are widely deployed in the AL field as the literature suggests [34], mainly due to their strong performance in terms of calculation efficiency and effectiveness in the process of selecting the most confusing instances. On the other hand, in the SSL field research works exist [8,35] proving the effectiveness of probabilistic iterative schemes. As the nature of these types of metrics is similar, they can prove to be a robust combination for the construction of schemes such as the proposed. Moreover, it is also known [36] that the SSL self-training technique further helps to overpass the lack of exploration problems that occur during the AL entropy-based training process causing the algorithms to stuck at suboptimal solutions, continuously selecting instances which do not improve the current classifier.

The proposed algorithm can be characterized as a simple yet very effective wrapper algorithm that can utilize a wide range of learners, assuming that they can produce probability distributions for their predictions. A detailed presentation of the algorithm follows in the next paragraphs.

Let ***D*** denote the initial training set, consisting of a labeled set of examples ***L*** and an unlabeled set of examples ***U*** thus defining a labeled ratio ***R***, as in the following equation:

(1) $$Labeled\;Ratio=\frac{size(L)}{size(L+U)}$$

where the size(X) function returns the size of a set of instances.

Initially, a base learner (***CLS***) is selected and trained on ***L***. Afterwards, a self-training scheme is employed with the aim to augment the ***L*** using the available unlabeled examples of ***D***. The number of unlabeled examples utilized in each iteration is conservatively selected taking in account the size of the initial labeled set, using also a control parameter ***T,*** setting the percentage of unlabeled examples related to the size of the initial labeled set. The number of maximum unlabeled instances selected in each iteration is calculated as follows:

(2) maxUnlabPerIter=T*R*size(D)

In each iteration (***i***), one of the two learning approaches is employed successively. The self-training loop terminates in a maximum number of iterations ***MaxIter*** or in the case of exhaustion of the pool of unlabeled examples.

Starting with the SSL round, the ***CLS*** is applied on the current unlabeled set ***U^i^*** and a matrix of predictions ***M_pr_*** is constructed along with the prediction probability for each unlabeled instance, resulting in a $size\left(U^{i}\right)\;x\;\left(l+2\right)$ dimensions matrix, where l+2 is the number of features, including the predicted labels and the corresponding prediction probabilities. The SSL round uses machine labeling in order to balance the expensive human effort and examination process required to label the data. The ***M_pr_*** is sorted descendingly utilizing the prediction probabilities while the rest of the ***maxUnlabPerIter*** elements are discarded. The ***maxUnlabPerIter*** instances along with their predicted labels are stored in ***M_final_***.

Following the method flow, an AL round is deployed in every other iteration. In this round, the algorithm attempts to construct a matrix containing the entropy estimation of each unlabeled instance ***EntrU^i^***. The base learner is applied on ***U^i^*** and the distribution of probabilities are exported in matrix ***DistU^i^*** of dimensions $size\left(U^{i}\right)\;x\;num\_classes\left(D\right)$, where the num_classes(X) function returns the number of classes of a dataset. Having produced ***DistU^i^***, the calculation of entropy estimation matrix is performed using the next formula, to compute each one of its elements (***j***):

(3) $$Entropy_{j}=\sum_{k=1}^{num\_classes(D)}-p_{k}*\log_{2}p_{k}$$

where $p_{k}$ denotes the probability of *k* class for instance ***j***, already contained in ***DistU^i^***.

Subsequently, ***EntrU^i^*** is sorted in descending order, as the most confusing examples, with entropy values near one, should be placed on the top of the matrix. The top ***maxUnlabPerIter*** instances are kept in ***EntrU^i^*** with the rest of them being discarded. Human expertise is utilized to label the ***maxUnlabPerIter*** instances and a matrix containing the human-labeled instances ***M_final_*** is constructed with the size of maxUnlabPerIter x (l+1), where l+1 is the number of features, including the class.

During each iteration, the ***M_final_*** instances are added to the current labeled set ***L^i^*** and removed from the current unlabeled set ***U^i^***. The ***CLS*** is re-trained at the start of each self-training iteration in order to be utilized again. When the termination criteria are met, the algorithm exits the self-training loop having constructed the augmented labeled set ***L^augmented^*** (≡***L^last iteration^***). As a final step, the ***CLS*** is trained on the augmented labeled set in order to be applied on the unknown test cases. The exact implementation of the combination scheme is presented in Algorithm 1.

## 4. Experimentation and Results

In order to examine the efficacy of the proposed scheme, an exhaustive experimentation procedure was followed. At first, fifty-five (55) benchmark datasets were extracted from the UCI repository [14], related to a wide range of classification problems. To further enhance the variance and complexity of the classification process, all datasets were partitioned and examined according to the resampling procedure of k-fold cross-validation [37]. Following the method’s steps, each subject dataset is shuffled and then divided into ***k*** unique data groups. By holding out one of the groups as a test set and utilizing the rest as a train set, ***k*** new datasets are generated. The ***k*** parameter was set equal to ten, as it is commonly selected by the majority of the literature.

The main aim of the experimentation process was to prove the superiority of the combination scheme against the competing methods of the supervised, semi-supervised and active learning using always the same amounts of labeled and unlabeled data under the same base learner model. In more detail, the supervised method is trained only on the initial labeled set while the semi-supervised rival method utilizes also the initial unlabeled set in the same manner that is also exploited in the proposed combination scheme. Moreover, as baseline AL opponent the random sampling [7] process is implemented in a similar way with the rest of the combination self-training procedure, also utilizing the initial unlabeled set.

For this purpose, all training subsets were further divided into two sets, an initial labeled set and an initial unlabeled set, using four different labeled ratios ***R***. As the initial datasets contained a hundred percent of the instance labels, in order to simulate the human expert labeling process, all the original labels for the constructed unlabeled sets were stored separately in order to be retrieved whenever the algorithm required to query the human expert. Thus, each original dataset was augmented into forty derived datasets. In detail, the ***R*** values were set to 10%, 20%, 30%, and 40%. As regards the proposed algorithm’s parameters, the control parameter ***T*** was set equal to 10%, while the ***MaxIter*** parameter was empirically selected equal to 10 in order to impose a maximum of 40%, in relation to the original dataset size, limit (can be calculated using Equation (2) multiplied by the ***MaxIter*** parameter of unlabeled instances for selection and augmentation of the initial labeled set in the case of ***R*** = 40%.

As a comparison measure, the average classification accuracy over each ***R*** was used. In order to draw general conclusions for the efficacy of the combination scheme, a wide range of classification models and meta-techniques were employed, incorporated in each one of the four learning methods. A brief description for each one of the base learners is presented:

• **BagDT**: In this model, the bootstrap aggregating (bagging) [38] meta-algorithm was applied along with the use of the C4.5 decision trees [39] classifier. The bagging technique is often adopted to reduce the variance and overfitting of a base learner and enhance its accuracy stability. The basic idea behind this technique is the generation of multiple training sets by uniformly sampling the original dataset.

• **5NN**: The k-nearest neighbors [40] classifier belongs to the family of lazy learning algorithms. By examining the k closest instances in a defined feature space, it classifies a given test instance by plurality voting on the labels of the k instances.

• **Logistic**: The logistic regression, also commonly referenced as the logit model, is a statistical model that utilizes the logistic function in order to model binary dependent variables, thus fitting very well with categorical targets. In problems where the target variable has more than two values, multinomial logistic regression is applied [41].

• **LMT**: The logistic model tree [42] classification model combines logistic regression with decision trees. The main idea behind the classifier is the use of linear regression models as leaves of a classification tree.

• **LogitBoost**: This classifier is a boosting model proposed by Friedman et al. [43]. It is based on the idea that the adaptive boosting [44] method can be thought as a generalized additive model, thus the cost function of logistic regression can be applied.

• **RF**: One of the most robust ML learners is the random forests [45] model, which is capable of tackling regression and classification problems. Its operation is based on the construction of multiple decision trees using random subsamples of the original feature space. The aggregation of the results is achieved via majority voting. Due to its inner architecture, it is known to efficiently handle the overfitting phenomena.

• **RotF**: The rotation forest model constitutes an ensemble [46] classifier proposed by Rodriguez and Kuncheva [47]. Following the flow of this algorithm, the initial feature space is divided in random subspaces. The default feature extraction algorithm applied to create the subspaces is the principal component analysis (PCA) [48], aiming to increase the diversity amongst the base learners.

• **XGBoost**: The extreme gradient boosted trees [49] algorithm, is a powerful implementation of gradient boosted decision trees. Under this boosting [50] scheme, a number of trees are built sequentially with each time the goal to reduce errors produced from the previous tree, thus each tree is fitted on the gradient loss of the previous step. The final decision is produced from the weighted voting of the trees. The XGBoost algorithm is a very scalable algorithm that has shown to perform very well on large datasets or sparse datasets utilizing parallel and distributed execution methods.

• **Voting (RF, RotF, XGBoost)**: As a last effort to further explore the potential of more complex classification models in the combination scheme, the construction of an ensemble classifier by majority voting the results of three of the most robust models: RF, RotF, and XGBoost was put forward. As regards the extraction of probabilities, the average of the exported probabilities for the three classifiers was considered as the best option.

The experimental results in terms of classification accuracy for each base learner are organized in Table 1, Table 2, Table 3, Table 4 and Table 5 and supplementary material Tables S1–S4, categorized according to the four label ratios (10%, 20%, 30%, 40%) for each learning method. The bold values in the tables indicate the highest accuracy for the corresponding dataset and the subject labeled ratio.

**Figure 3:**
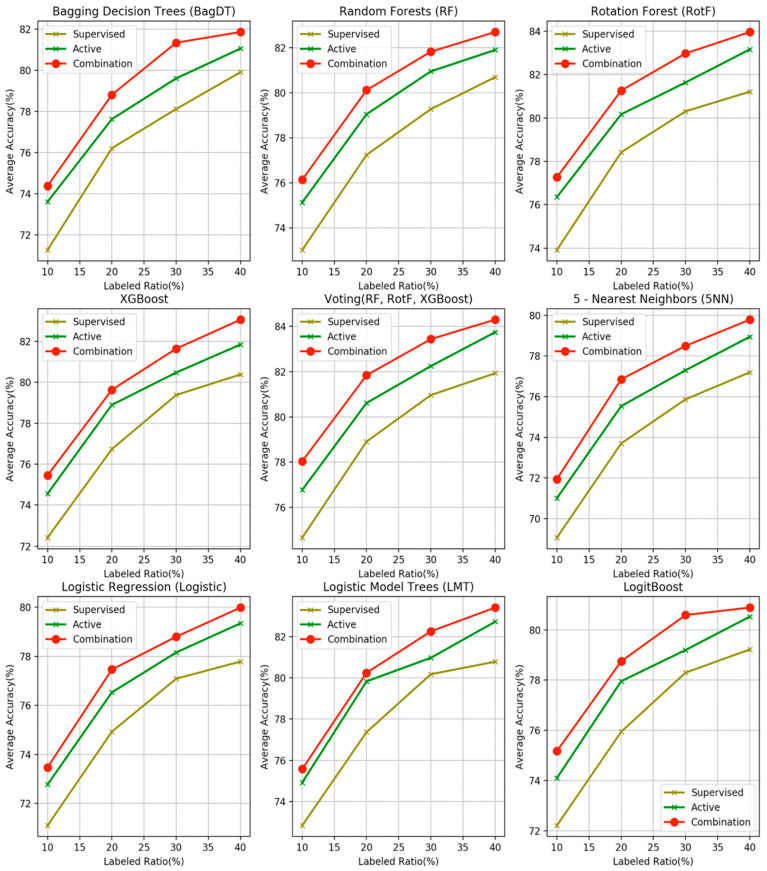
Performance comparison of the proposed combination scheme, in terms of average accuracies over fifty-five datasets and four labeled ratios, against the corresponding methods of active learning (AL) and supervised learning (SL) for the nine base classifiers.

The superiority of the proposed combination scheme regarding the classification accuracy is prominent. The following important observations are derived from the accuracy tables:

- The proposed combination method outperforms all other four learning methods in all four labeled ratios and for all the nine base learners used as control methods, in terms of average accuracy. This argument is also validated in Figure 3, where the comparisons are visually assembled and a progressive picture of the performance of the two dominant methods is presented as the ***R*** increases. The SL method was also included as a baseline performance metric.
- It is observed by the accuracy tables that the proposed method steadily produces significantly more wins on each individual dataset through all the experiments carried out.

Following the accuracy examination, the Friedman aligned ranks test [51] was conducted. In Table 6, Table 7, Table 8, Table 9, Table 10, Table 11, Table 12, Table 13 and Table 14, the results of the statistical tests for each one of the nine base learners divided into the four labeled ratios used, are presented. These lead to the following assumptions:

- The non-parametric tests assess the null hypothesis that the means of the results of two or more of the compared methods are the same by calculating the related *p*-value. This hypothesis can be rejected for all the nine algorithms and for all labeled ratios as all calculated *p*-values are significantly lower than the significance level of *a = 0.10*.
- Moreover, the Friedman rankings confirm that for all nine base learners and regardless of the labeled ratio, the proposed combination scheme ranks first ahead of all other learning methods in coincidence with the accuracy experimental results.

Since the Friedman test null hypothesis was rejected, the Holm’s [52] post-hoc statistical test was also applied with an alpha value of 0.10. The aim of the Holm’s test is to detect the specific differences between the combination scheme and the other learning methods, thus the null hypothesis under evaluation is that the mean of the results of the proposed method and against each other group is equal (compared in pairs). The post-hoc results are also presented in the corresponding ranking test tables for each one of the base learners. By observing the adjusted *p*-values of the Holm’s tests, it is concluded that:

- The proposed combination method performs significantly better on 105 of the total 108 compared method variations for the nine base learners over the four labeled ratios.
- The AL methods for the Logistic, the LMT and the LogitBoost classifiers accept the mean significant difference test for one label ratio each, 30%, 20%, 40% accordingly. However, the adjusted *p*-values show small differences over the alpha of 0.10.

Summarizing the test results, both Friedman Aligned Ranks tests and Holm’s one vs all comparison tests verify the superior performance of the proposed method over a wide range of scenarios and algorithm comparisons. 

**Figure 4:**
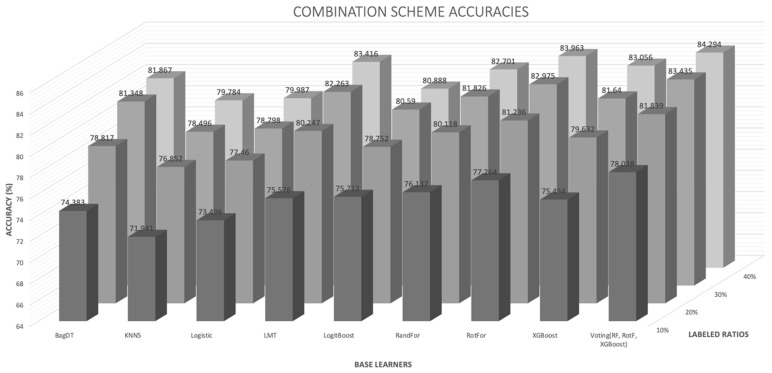
Average accuracies for the proposed combination scheme regarding different base learners and labeled ratios.

To better observe the individual results regarding the combination schemes and the role of the base learners incorporated, the average accuracies were plotted in Figure 4. The outcome was as expected the following: The ensemble voting (RF, RotF, XGBoost) classifier outperforms the rest models in all labeled ratios. As the first indication of such an outcome, the improved prediction probabilities derived from the averaging of the three classifier probabilities, on which the combination scheme relies, it would be a promising starting point for seeking a robust proof to strictly explain the performance boost. Thus, on the one hand, the most confusing unlabeled instances, through the entropy calculation, and on the other hand, the most confident unlabeled instances, through the distribution of prediction probabilities, are detected using the distribution of prediction probabilities. Such behaviors seem to also emerge in other relevant ensemble wrapper algorithms [53].

## 5. Modification

Pointing towards the improvement of the proposed method, it is obvious by the statistical analysis and ranking results that a slight increase in the performance of the SSL part could have a significant impact on the overall efficiency of the combination scheme.

**Figure 5:**
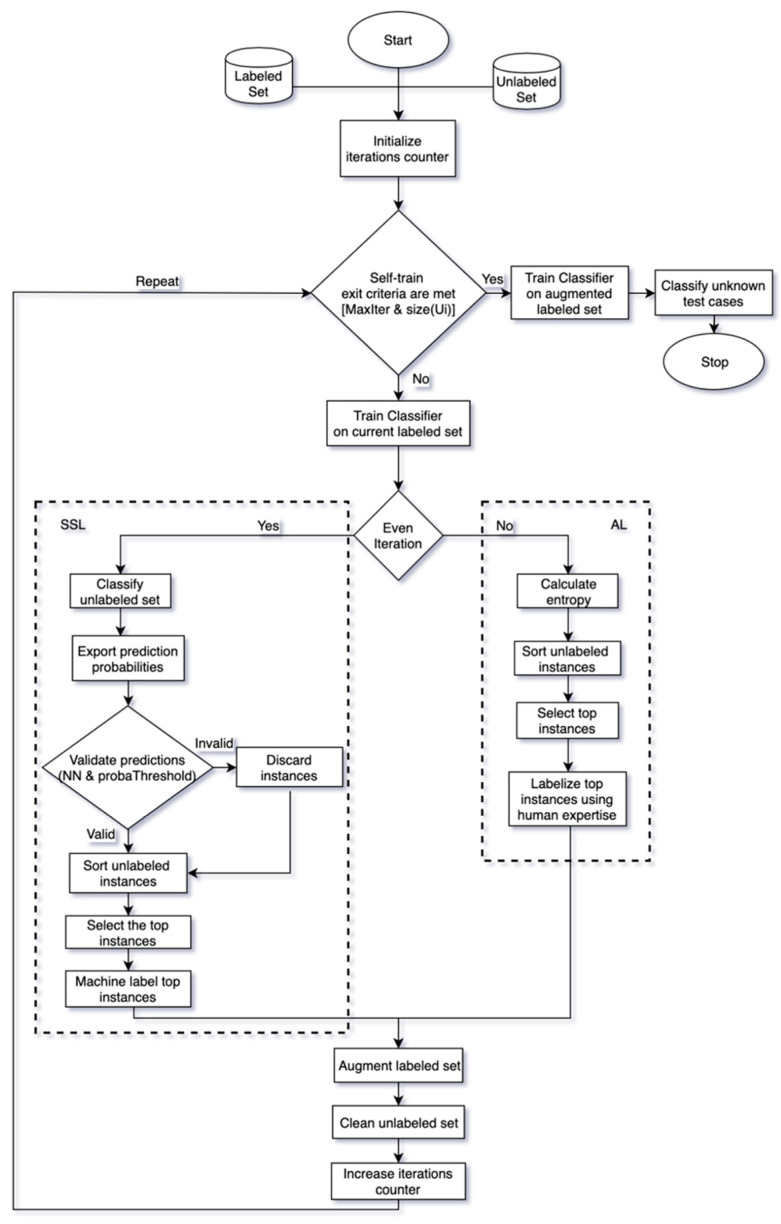
Graphical abstract of the proposed combination framework after the introduction of the semi-supervised learning (SSL) improvements.

In this direction, careful observation of the execution of the proposed algorithm revealed the weakness of the SSL prediction probabilities, which, in many cases, leads to the selection of the wrong instances to be labeled. In order to augment the probabilistic information available for the proposed method, as regards the SSL part, a lazy classifier (kNN) was integrated into the instance selection process. Such a development, on the one hand, augments the proposed method with a second view of the labels for the unlabeled set, and on the other hand, does not significantly increase the computational overhead as this family of classifiers does not need training. As a second measure to strengthen the SSL instance selection criteria, the empirical approach of setting a lower limit on the minimum accepted probability for an unlabeled instance was adopted using the formula:

(4) $$probaThreshold=\frac{num\_classes(D)+1}{2*num\_classes(D)}$$

whereby utilizing the num_classes(X) function the dependence on the dataset characteristics is lifted. A more compact representation of the SSL part modifications is given in Algorithm 2, while the abstract flow chart of the improved combination framework is presented in Figure 5.

**Algorithm 2:** SSL modification
10:[Execute Algorithm 1 steps (until Alg. 1 line 10)]11: IF i modulo 2 == 0:12:  SET ***probaThreshold* = [**num_classes(***D***) + 1]/[2 * num_classes(***D***)**]**13:  SET the number of nearest neighbors ***numNeib***14:  INITIALIZE the ***NN*** classifier on ***L^i^*** using ***numNeib***15:  16:  Classify(***U^i^***) using ***CLS*** and construct matrix ***M_pr_*** containing corresponding prediction probabilities along with the predicted labels17:  FOR_EACH ***instance*** of ***M_pr_***:18:   IF cls_predicted_class(***instance***) != nn_predicted_class(***instance***)    OR cls_probability(***instance***) < ***probaThreshold***:19:    DISCARD ***instance*** from ***M_pr_***20:   END_IF21:  END_FOR_EACH22:  SORT ***M_pr_*** descending according to the prediction probabilities23:  STORE the top ***maxUnlabPerIter*** instances of ***M_pr_*** in a matrix ***M_final_***24:  /* now containing the most confident instances along with their predictions */25:[Continue Algorithm 1 steps (from Alg. 1 line 16)]


The improved combination scheme was further tested against the most robust AL frameworks found in the literature. In detail, the query strategies of least confidence (LC), margin sampling (MS) and entropy sampling (ES) were considered to be compared with the modified proposed scheme. The major aspects concerning these strategies [7] follow below.

• **LC**: The objective of this strategy is to identify the least confident unlabeled instances by examining the probability of the most probable label for each unlabeled instance. The strategy continues by selecting the instances having the lowest probable labels and presents them to the human expert to be labeled in order to augment the initial labeled set.

• **MS**: As an improvement of the LC strategy, MS attempts to overcome the disadvantageous selection process of only considering the most probable labels by calculating the differences of the most probable and the second most probable label for an unlabeled instance. Afterwards, those calculated differences are sorted and the instances with the lowest differences are selected to be labialized.

• **ES**: This strategy, part of which is also integrated into the AL counterpart of the proposed scheme, computes the entropy measure (similar to Equation (3)) for each unlabeled instance using the distribution of prediction probabilities. The most entropic instances are then selected to be displayed to the human expert in order to enlarge the original labeled set.

**Figure 6:**
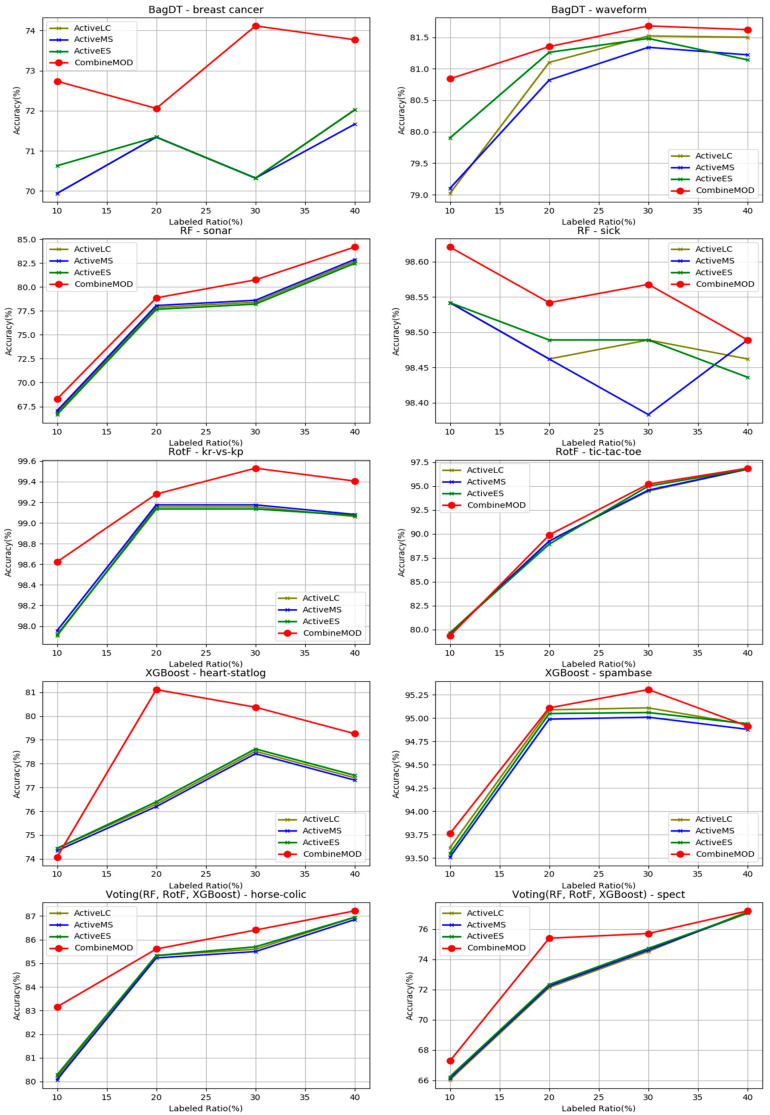
Progression of accuracies for the modified combination scheme against the four AL strategies (least confidence (LC), margin sampling (MS), entropy sampling (ES)) for five different base learners on ten different benchmark datasets.

In Figure 6, ten experiments display the performance comparison in terms of classification accuracy regarding the three AL methods against the modified combination scheme. The experiments are categorized by the five base learner models that were integrated into the methods. In each experiment, a different benchmark dataset was deployed using four different ***R****s* equal to 10%, 20%, 30%, and 40% accordingly.

The experimental results confirm the efficiency of the modified combination scheme against the AL methods. It can be extracted from the figure that the proposed technique in all ten cases performs equally or better from its rivals’ accuracies. Moreover, the figure suggests that the three AL methods produce closely related accuracy results, as in four of the ten test cases, their performance was almost identical. The previous outcome can be explained by exploring the metrics utilized in these strategies, which are all derived from the prediction probabilities of the base learners.

Closing this section, in the conducted experiments on real-world benchmark datasets, the proposed combination scheme was compared with the SL, the SSL, and the AL methods. The experiments show that the proposed method outperforms the compared methods. Therefore, in the future, it is very important to conduct more insightful theoretical analyses on the effectiveness of the proposed approach and explore other appropriate selection criteria for filtering the informative unlabeled instances, in order to generalize the results with more confidence.

## 6. Conclusions

In this research work, a new wrapper algorithm was proposed combining the AL and SSL methods with the aim of efficiently utilizing the available unlabeled data. A plethora of experiments was conducted for evaluating the efficacy of the proposed algorithm in a wide range of benchmark datasets against other learning methods using a variety of classifiers as base models. In addition, four different labeled ratios were investigated. The proposed algorithm prevails over the other learning methods as statistically confirmed by the Friedman aligned ranks non-parametric tests and the Holm’s post-hoc tests. To further promote the use of the proposed algorithm, a software package was developed while more details about this package can be obtained from the link found in the Appendix A.

Regarding the performance boost that was experimentally observed while applying the proposed combination scheme on the numerous datasets, there is strong evidence that the vigorous AL method can efficiently improve its performance utilizing SSL schemes such as the self-training technique. Even in cases were the individual SSL method was not performing dexterously; when integrated in the AL and SSL proposed wrapper the performance of the overall scheme was significantly improved compared to the plain AL method. Moreover, in the case that the majority of the instances used in a learning scheme are automatically labeled, the performance may be unsatisfactory, and in some cases, it may even be worse than the SL baseline accuracy. For this reason, a fundamental requirement arises; that of defining a sufficient threshold of human expert intervention on the labeling process to successfully combine AL and SSL methods. Such a fine-tuning process is criticized as highly application-specific and challenging to automate. Furthermore, it can be noticed by the results, that on datasets with very small initial labeled sets, the proposed scheme can be beneficiary as the initially learned decision boundaries of such datasets can be possibly inaccurate, thus unlabeled instances near these boundaries could be falsely classified. This is an implication that the AL part of the proposed scheme could efficiently tackle.

For future work, a number of areas have been identified and are worth exploring as they seem promising in the direction of improving the classification abilities of the proposed algorithm. As a major first research area, that is expected to have a high impact on the combination scheme’s performance in terms of accuracy and execution time would be the investigation of different instance selection strategies than those that are currently employed. In the AL part of the proposed algorithm, two common alternatives are the least confidence [54] and the margin sampling [55] algorithms, which utilize the unlabeled data under a different scope. Moreover, more complex query scenarios than the plain pool-based sampling used, like query synthesis [56] could also be beneficial. As regards the semi-supervised part, simple techniques like the integration of weights annotating the instances assessed as informative by the SSL part of the algorithm could further improve the overall accuracy of the combination scheme as suggested in [35,57].

Another interesting research area would be that of the extreme outlier detection algorithms. The incorporation of such algorithms in the proposed algorithm would have an immediate impact on the quality of the selected candidate unlabeled instances that are used to augment the labeled set in each self-training iteration, thus resulting in more robust inner models. A few of the very well-known techniques that could be directly implemented in the combination scheme are the local outlier factor [58] for detecting anomalous values based on neighboring data or the isolation forest [59], which is a tree-based outlier detector.

Other research areas that would bear further improvement to the proposed algorithm include preprocessing algorithms, for instance, PCA for dimensionality reduction and production of more informative features or other feature selection techniques such as univariate feature selection [60]. Speaking of the integrated base learners, the introduction of online learners like the Hoeffding adaptive tree [61] and Pegasos [62] or deep learning architectures based on deep neural networks [63] and deep ensembles [64] could make the proposed algorithm sufficient for tackling streaming and big data problems.

Finally, by combining schemes from the fields of active regression learning [65,66] and semi-supervised regression [53] along with the proposed classification algorithm, a general combination scheme could be put forward that would be able to handle numeric and categorical targets.

## Figures and Tables

**Table 1:** Classification accuracies of bagging-decision trees (BagDT) on four different ratios.

		*R* = 10%				*R* = 20%				*R* = 30%				*R* = 40%			
	Method	Supervised	Semi-Supervised	Active Random	Combination	Supervised	Semi-Supervised	Active Random	Combination	Supervised	Semi-Supervised	Active Random	Combination	Supervised	Semi-Supervised	Active Random	Combination
Dataset																	
anneal		88.866	88.524	93.211	**94.660**	95.210	95.988	96.770	**99.107**	96.659	96.547	97.551	**98.663**	97.773	97.215	98.326	**98.885**
arrhythmia		58.425	57.981	62.614	**63.285**	65.063	63.314	68.807	**69.691**	70.140	70.589	69.483	**70.812**	70.348	69.498	73.242	**73.464**
audiology		52.253	50.474	56.166	**56.640**	61.087	61.482	65.593	**68.636**	65.138	64.684	73.004	**74.308**	71.660	70.791	75.178	**80.079**
autos		45.381	44.286	**50.167**	48.286	52.119	56.167	**61.000**	55.619	60.976	58.143	63.405	**66.381**	65.405	59.048	69.738	**72.690**
balance-scale		73.103	75.832	74.560	**75.996**	76.487	75.540	80.476	**81.608**	81.129	78.571	**81.941**	81.764	83.372	78.093	**83.518**	83.372
breast-cancer		69.926	**70.628**	70.603	69.544	69.963	70.616	70.603	**70.961**	70.961	70.616	70.591	**71.675**	71.293	70.616	70.948	**74.803**
bridges-version1		**45.727**	**45.727**	**45.727**	**45.727**	53.273	**57.909**	57.273	56.909	60.000	60.727	60.000	**62.455**	61.000	61.091	**64.909**	62.909
bridges-version2		**43.000**	**43.000**	**43.000**	**43.000**	48.455	51.091	**59.818**	53.909	60.000	**64.000**	63.091	62.818	62.091	59.182	**63.636**	62.000
clevalend		**78.215**	76.559	75.849	75.559	73.247	74.570	**79.204**	78.194	79.516	76.839	80.462	**81.484**	**83.151**	78.516	80.495	81.806
cmc		48.066	48.329	49.356	**49.691**	50.302	51.455	**54.307**	51.260	50.576	52.339	51.527	**53.154**	53.220	**54.100**	52.674	53.428
column_2C		76.452	75.806	75.806	**80.645**	80.323	80.323	80.323	**84.516**	81.613	80.968	82.258	**82.903**	82.258	82.258	82.903	**83.226**
column_3C		**79.032**	**79.032**	77.419	77.742	79.355	78.710	79.677	**83.226**	80.323	80.645	78.065	**83.871**	79.355	79.032	82.258	**82.903**
credit-rating		84.783	83.768	85.217	**85.362**	84.638	84.783	85.217	**85.507**	85.652	85.507	85.652	**86.667**	85.072	85.942	86.087	**86.232**
cylinder-bands		58.333	57.037	57.222	**59.444**	59.630	60.000	59.259	**60.556**	58.889	58.704	58.148	**59.074**	**60.556**	57.963	58.519	59.259
dermatology		71.089	70.578	82.770	**87.447**	84.977	83.348	91.036	**95.931**	91.006	91.029	93.483	**95.113**	93.461	89.647	92.658	**96.742**
ecoli		67.282	68.173	**76.471**	76.194	79.144	76.185	80.936	**81.230**	80.936	80.963	81.827	**83.324**	80.348	79.162	**83.324**	83.316
flags		**50.053**	48.553	48.526	49.921	51.105	51.079	52.026	**52.579**	55.658	52.184	51.132	**56.763**	**56.237**	52.605	55.289	52.711
german_credit		70.500	69.200	69.300	**70.800**	70.900	69.200	69.500	**71.400**	69.500	70.500	**74.000**	**74.000**	**73.600**	71.800	72.500	73.000
glass		50.498	47.684	51.991	**53.810**	64.524	59.848	57.468	**65.952**	60.303	60.779	**67.727**	67.338	**69.113**	64.372	67.229	**69.113**
haberman		**72.538**	71.591	71.559	70.882	72.860	73.204	70.882	**73.860**	71.871	72.538	72.204	**73.194**	**71.871**	71.538	71.839	71.237
heart-statlog		72.963	71.481	74.444	**75.185**	75.556	77.037	78.148	**80.741**	77.778	76.667	80.370	**84.074**	79.630	77.037	82.222	**82.963**
hepatitis		80.083	**81.958**	79.375	81.917	78.792	80.083	80.000	**81.875**	**82.542**	**82.542**	79.958	79.417	**82.542**	80.000	78.000	79.875
horse-colic		78.551	78.544	82.605	**84.219**	84.775	83.138	82.868	**85.308**	83.401	82.590	84.219	**85.300**	83.949	85.030	85.571	**85.841**
hungarian-heart		81.000	78.632	78.310	**82.391**	**81.023**	80.701	78.276	78.966	78.621	78.644	78.977	**83.057**	76.897	76.885	**81.655**	79.310
hypothyroid		98.357	98.199	98.808	**99.602**	99.072	98.887	99.046	**99.602**	99.099	99.099	99.417	**99.549**	99.285	99.311	99.443	**99.576**
ionosphere		75.802	77.802	**86.643**	84.667	88.040	88.032	88.333	**92.317**	86.619	86.357	91.183	**92.317**	90.611	89.746	90.611	**92.032**
iris		72.667	77.333	84.667	**86.667**	88.000	89.333	**90.667**	**90.667**	**93.333**	**93.333**	91.333	92.000	**93.333**	**93.333**	**93.333**	92.667
kr-vs-kp		95.025	95.244	96.340	**98.499**	97.027	97.310	98.091	**99.249**	97.998	98.216	98.748	**99.343**	98.998	98.811	99.218	**99.343**
labor		**66.333**	**66.333**	**66.333**	**66.333**	65.667	**70.333**	70.000	66.333	70.000	73.667	77.000	**87.667**	77.000	**79.000**	78.667	**79.000**
letter		77.955	77.235	81.320	**84.490**	83.775	82.470	86.570	**89.840**	86.570	85.770	89.165	**92.175**	88.335	86.375	90.280	**92.620**
lymphography		65.476	66.143	**68.952**	67.619	**77.571**	72.381	71.571	71.667	72.905	70.857	75.571	**79.048**	74.286	76.238	77.619	**79.667**
mushroom		99.163	99.323	99.729	**100.000**	99.877	99.889	99.914	**100.000**	99.951	99.902	99.975	**100.000**	99.963	99.975	99.975	**100.000**
optdigits		89.021	86.174	90.783	**92.829**	92.189	90.036	93.043	**95.979**	92.936	90.587	93.719	**95.712**	94.288	90.854	94.555	**95.498**
page-blocks		95.414	95.468	95.980	**97.058**	96.236	95.926	96.547	**97.113**	96.620	96.583	**97.369**	97.241	**97.168**	97.004	97.150	**97.168**
pendigits		93.122	92.849	94.796	**96.516**	95.515	94.860	96.061	**97.862**	96.179	96.006	96.871	**98.335**	96.880	96.243	97.362	**98.071**
pima_diabetes		71.880	73.055	**75.135**	74.479	**75.911**	73.970	74.747	73.445	75.270	75.261	73.841	**76.307**	**76.309**	76.044	74.498	75.930
postoperative		**65.556**	**65.556**	**65.556**	**65.556**	67.778	**71.111**	64.444	66.667	62.222	**67.778**	66.667	**67.778**	64.444	**67.778**	65.556	**67.778**
primary-tumor		29.474	30.339	**35.954**	34.795	35.651	34.750	**38.610**	36.248	**38.610**	34.198	37.745	37.469	40.401	35.677	**41.578**	38.930
segment		90.736	90.779	91.472	**94.329**	93.420	93.117	94.372	**97.229**	93.853	94.502	95.455	**97.359**	94.589	94.892	96.017	**97.186**
sick		97.640	97.587	97.932	**98.648**	98.038	98.118	98.117	**98.754**	98.118	98.144	98.223	**98.728**	98.277	98.144	98.595	**98.621**
solar-flare		67.914	64.807	**68.439**	68.164	69.790	68.941	70.081	**70.322**	70.115	70.538	**71.532**	71.260	70.336	71.536	71.448	**71.905**
sonar		59.524	60.976	**64.881**	60.548	66.833	64.429	**68.714**	67.881	69.214	70.595	**74.048**	71.119	70.714	69.214	**76.476**	75.024
soybean		64.241	61.624	**75.980**	70.119	79.211	75.835	84.633	**86.091**	84.486	82.564	87.551	**90.635**	86.520	84.318	91.355	**93.116**
spambase		89.937	90.285	90.545	**92.827**	91.328	91.784	92.349	**94.305**	92.132	92.827	92.805	**94.631**	92.741	92.067	93.219	**94.610**
spect		61.316	61.494	61.864	**63.367**	**68.506**	65.344	66.099	66.902	66.717	71.978	73.390	**80.342**	**79.186**	74.895	77.025	76.084
sponge		**86.250**	**86.250**	**86.250**	**86.250**	91.071	**92.500**	**92.500**	**92.500**	**92.500**	**92.500**	**92.500**	**92.500**	**93.750**	92.500	92.500	**93.750**
tae		32.375	34.375	**40.417**	38.375	38.333	36.958	38.333	**41.000**	42.958	41.708	40.333	**52.250**	44.375	42.333	53.583	**54.875**
tic-tac-toe		70.254	70.985	74.221	**76.620**	78.189	74.326	80.895	**83.091**	82.467	76.404	84.864	**86.951**	84.444	80.684	89.457	**91.864**
vehicle		64.189	62.289	67.150	**67.615**	66.916	67.134	**70.445**	69.618	70.221	70.091	**73.175**	71.161	**73.060**	70.454	72.108	72.696
vote		94.049	94.952	94.276	**95.174**	95.412	95.418	94.049	**96.321**	95.412	95.640	**96.327**	95.872	95.640	95.645	**96.781**	96.327
vowel		48.283	49.192	52.424	**54.040**	60.808	60.707	67.980	**70.202**	70.707	65.758	74.545	**77.172**	73.939	68.485	80.303	**83.737**
waveform		78.020	75.600	79.180	**79.700**	80.300	77.540	79.160	**81.340**	79.160	78.380	81.200	**81.260**	80.700	78.160	80.880	**81.740**
wine		74.314	78.203	80.425	**84.935**	88.758	85.425	91.601	**93.268**	90.458	89.379	93.301	**96.078**	92.157	91.601	92.712	**94.379**
wisconsin-breast		91.986	92.418	92.416	**95.422**	94.559	93.704	94.994	**96.422**	95.565	94.277	95.565	**95.994**	94.996	95.277	95.137	**96.137**
zoo		**57.455**	**57.455**	**57.455**	**57.455**	75.273	75.273	78.273	**85.182**	81.273	82.273	86.273	**88.273**	84.273	86.273	88.273	**93.182**
**Average**		71.270	71.158	73.611	**74.383**	76.216	75.847	77.631	**78.817**	78.125	77.863	79.614	**81.348**	79.913	78.623	81.062	**81.867**

**Table 2:** Classification accuracies of random forests (RF) on four different ratios.

		*R* = 10%				*R* = 20%				*R* = 30%				*R* = 40%			
	Method	Supervised	Semi-Supervised	Active Random	Combination	Supervised	Semi-Supervised	Active Random	Combination	Supervised	Semi-Supervised	Active Random	Combination	Supervised	Semi-Supervised	Active Random	Combination
Dataset																	
anneal		83.740	83.965	85.414	**87.976**	87.418	87.194	90.979	**92.203**	90.869	90.087	92.985	**93.762**	92.875	92.427	93.871	**94.871**
arrhythmia		56.638	56.633	60.406	**60.623**	59.720	58.841	60.396	**64.599**	59.285	58.184	63.275	**64.821**	62.377	59.517	63.937	**65.488**
audiology		42.490	36.739	**53.518**	45.652	56.700	54.466	**63.794**	59.308	61.976	63.241	**72.134**	68.617	68.123	67.767	**73.874**	73.379
autos		48.833	49.714	**52.119**	51.238	56.929	57.452	65.381	**66.286**	68.286	69.262	70.667	**74.595**	68.738	69.738	77.976	**79.476**
balance-scale		78.067	77.588	79.027	**80.461**	79.519	78.241	79.841	**80.161**	82.084	79.524	**82.087**	81.935	**82.081**	80.806	81.910	81.129
breast-cancer		69.249	70.283	67.131	**72.044**	67.180	**68.559**	64.310	68.276	66.773	**68.153**	67.192	66.441	67.106	**69.212**	64.754	68.596
bridges-version1		**44.636**	**44.636**	**44.636**	**44.636**	44.636	38.000	**46.545**	44.727	46.636	44.545	**52.545**	45.545	51.545	45.545	52.273	**55.000**
bridges-version2		**41.818**	**41.818**	**41.818**	**41.818**	45.545	37.000	**49.273**	46.455	48.364	40.000	**53.182**	45.545	**53.273**	47.545	53.182	52.273
clevalend		76.849	75.172	**79.505**	78.817	80.161	79.495	**83.430**	81.108	81.086	79.774	**82.806**	81.774	82.452	82.108	**83.774**	81.806
cmc		48.881	48.406	**50.102**	48.401	49.964	51.322	**51.798**	50.239	51.525	51.730	50.914	**52.544**	51.116	52.000	50.908	**52.200**
column_2C		77.097	76.774	77.419	**79.355**	80.000	79.677	80.968	**81.290**	81.935	81.935	83.548	**83.871**	83.871	81.613	82.581	**85.484**
column_3C		77.742	79.032	79.677	**82.258**	82.903	82.258	**84.516**	82.903	82.581	83.226	81.290	**84.839**	81.935	82.258	81.290	**84.194**
credit-rating		82.319	82.464	83.478	**85.217**	83.768	84.203	84.493	**85.797**	83.768	84.203	84.928	**85.362**	84.638	84.348	84.783	**86.087**
cylinder-bands		59.259	58.519	63.333	**64.259**	65.185	59.444	**67.778**	65.741	67.593	60.185	**70.185**	70.000	67.222	60.000	**71.296**	70.185
dermatology		78.709	79.797	86.877	**92.598**	90.961	89.595	94.805	**95.616**	93.716	93.724	95.090	**96.456**	95.638	94.264	93.986	**96.179**
ecoli		74.091	72.906	77.674	**78.574**	80.339	77.968	84.189	**84.777**	84.189	80.651	84.225	**85.695**	83.307	84.216	85.410	**86.292**
flags		46.447	41.789	44.395	**48.421**	**52.763**	38.711	52.711	51.053	**54.737**	45.921	50.132	52.184	54.289	47.000	**57.789**	52.132
german_credit		72.900	72.200	**73.300**	72.700	74.000	73.600	73.900	**75.600**	73.900	73.700	**75.300**	**75.300**	74.900	71.600	74.500	**75.400**
glass		55.216	53.333	**57.987**	55.216	66.385	66.840	**68.701**	68.247	68.723	69.156	71.948	**75.714**	72.381	67.706	**74.762**	74.286
haberman		62.043	65.656	**66.344**	64.258	68.591	**69.903**	67.269	68.581	66.978	67.634	66.355	**69.237**	67.022	**68.989**	66.301	66.677
heart-statlog		74.074	75.185	74.815	**77.037**	78.519	78.519	78.148	**81.111**	79.259	78.519	81.481	**83.333**	80.741	78.889	**82.222**	81.852
hepatitis		80.667	80.708	80.125	**83.792**	78.167	79.458	81.250	**83.125**	82.000	82.542	78.667	**84.458**	81.917	81.875	78.667	**85.125**
horse-colic		79.069	79.595	79.857	**82.590**	84.227	83.664	83.949	**84.767**	83.686	82.057	84.219	**85.578**	83.941	85.856	84.767	**86.119**
hungarian-heart		79.276	79.264	79.989	**82.000**	**83.046**	**83.046**	81.000	82.011	**81.345**	**81.345**	80.287	81.000	81.644	**81.655**	81.299	80.966
hypothyroid		95.733	95.520	96.660	**99.258**	97.880	97.429	98.569	**99.444**	98.330	98.039	98.966	**99.391**	98.993	98.596	98.914	**99.364**
ionosphere		81.802	80.381	86.929	**88.603**	90.603	89.746	90.889	**92.889**	91.460	90.603	92.889	**93.460**	91.746	91.746	93.175	**94.032**
iris		83.333	83.333	88.000	**90.000**	93.333	92.000	**96.000**	94.667	**96.000**	95.333	**96.000**	94.667	**95.333**	**95.333**	94.667	**95.333**
kr-vs-kp		95.776	94.900	96.120	**98.623**	96.778	96.621	97.497	**99.280**	97.559	97.465	98.248	**99.406**	98.154	98.216	98.937	**99.312**
labor		**65.000**	**65.000**	**65.000**	**65.000**	73.667	69.667	**82.000**	80.333	82.000	77.000	**84.000**	82.667	**84.000**	73.333	82.667	82.333
letter		84.795	84.210	87.940	**90.915**	89.845	89.625	92.215	**95.300**	92.215	91.870	94.050	**96.290**	93.415	92.955	95.010	**96.435**
lymphography		68.238	67.571	73.619	**75.000**	74.190	76.286	**77.619**	76.238	74.333	76.333	**81.667**	80.333	79.048	77.619	**85.143**	85.095
mushroom		99.741	99.766	99.914	**100.000**	99.963	99.963	**100.000**	**100.000**	99.988	99.988	99.975	**100.000**	**100.000**	99.988	**100.000**	**100.000**
optdigits		94.573	94.431	95.765	**97.473**	96.406	96.050	97.011	**98.327**	97.135	96.762	97.438	**98.327**	97.438	96.886	97.865	**98.363**
page-blocks		95.870	95.797	96.218	**97.351**	96.492	96.455	96.675	**97.497**	96.748	96.766	97.004	**97.552**	96.985	96.912	97.479	**97.533**
pendigits		97.143	96.980	97.607	**98.463**	98.008	97.844	98.317	**99.236**	98.372	98.353	98.772	**99.245**	98.672	98.435	98.917	**99.172**
pima_diabetes		74.354	72.404	74.231	**74.612**	75.656	74.614	75.921	**76.304**	**77.092**	76.832	75.930	74.879	75.531	75.138	74.624	**76.048**
postoperative		**68.889**	**68.889**	**68.889**	**68.889**	62.222	66.667	62.222	**67.778**	63.333	64.444	64.444	**65.556**	60.000	**65.556**	63.333	63.333
primary-tumor		33.601	34.768	**37.424**	33.592	37.406	38.592	**43.039**	40.383	43.039	40.348	42.736	**43.913**	41.551	40.642	**43.627**	43.039
segment		93.333	93.463	94.372	**96.537**	95.281	95.238	95.844	**97.965**	95.628	95.671	96.970	**98.009**	96.537	96.364	97.403	**98.225**
sick		96.527	96.394	96.659	**98.595**	97.455	97.455	97.773	**98.542**	97.667	97.534	97.958	**98.462**	97.905	97.826	98.144	**98.436**
solar-flare		66.769	65.990	69.098	**69.375**	**70.512**	70.059	70.131	70.464	70.073	70.037	69.758	**71.550**	70.041	69.681	70.513	**72.590**
sonar		62.024	64.024	67.333	**69.643**	69.238	69.738	75.929	**79.333**	74.976	75.976	76.929	**81.238**	79.786	75.952	82.238	**82.690**
soybean		67.199	64.429	**76.281**	75.251	79.066	77.006	85.211	**87.990**	86.816	84.474	89.744	**92.822**	88.286	88.572	91.360	**93.110**
spambase		92.871	92.566	93.088	**94.588**	93.523	93.305	94.197	**95.544**	94.305	94.110	94.675	**95.675**	94.523	94.327	94.936	**95.588**
spect		69.270	**69.825**	68.522	69.270	72.395	72.133	71.284	**78.837**	75.582	71.948	78.159	**79.193**	**80.196**	77.765	79.895	77.381
sponge		**92.500**	**92.500**	**92.500**	**92.500**	**92.500**	**92.500**	**92.500**	**92.500**	**92.500**	**92.500**	**92.500**	**92.500**	**93.750**	**93.750**	92.500	**93.750**
tae		36.375	37.750	**41.750**	41.667	39.708	37.042	41.667	**43.542**	40.375	39.083	49.042	**49.625**	46.333	45.042	56.333	**57.667**
tic-tac-toe		76.512	74.942	78.396	**83.296**	81.735	80.171	84.343	**91.544**	84.240	81.110	88.515	**95.510**	89.764	82.156	91.549	**96.658**
vehicle		65.132	64.311	**70.339**	69.151	71.761	70.104	70.227	**73.167**	70.340	70.697	**74.115**	73.527	71.992	71.169	**74.591**	73.161
vote		94.503	94.276	94.958	**96.327**	95.196	94.503	94.947	**96.332**	95.412	95.180	**96.786**	96.332	96.559	96.327	**97.014**	96.781
vowel		34.444	33.737	**44.848**	42.727	56.566	55.657	**71.313**	71.111	71.010	69.899	84.545	**88.788**	80.808	80.000	91.616	**96.162**
waveform		83.660	82.940	**84.340**	83.920	84.300	84.140	84.140	**84.620**	84.140	83.860	84.440	**84.840**	85.100	83.960	**85.220**	84.840
wine		86.046	86.601	85.980	**94.412**	94.379	96.601	95.000	**98.301**	96.111	96.667	97.222	**98.333**	**98.333**	97.222	97.222	98.301
wisconsin-breast		94.414	94.986	95.130	**97.280**	96.135	95.418	96.420	**96.851**	**96.706**	96.565	96.280	96.565	95.994	96.280	96.280	**96.422**
zoo		**75.273**	**75.273**	**75.273**	**75.273**	79.273	77.273	79.273	**88.182**	85.273	79.273	87.273	**93.182**	88.273	84.273	87.273	**92.182**
**Average**		73.015	72.730	75.130	**76.137**	77.238	76.316	79.047	**80.118**	79.274	78.255	80.954	**81.826**	80.694	79.436	81.901	**82.701**

**Table 3:** Classification accuracies of rotation forest (RotF) on four different ratios.

		*R* = 10%				*R* = 20%				*R* = 30%				*R* = 40%			
	Method	Supervised	Semi-Supervised	Active Random	Combination	Supervised	Semi-Supervised	Active Random	Combination	Supervised	Semi-Supervised	Active Random	Combination	Supervised	Semi-Supervised	Active Random	Combination
Dataset																	
anneal		85.185	86.075	86.859	**91.080**	88.206	91.541	93.211	**94.206**	93.432	93.543	95.104	**96.215**	94.878	94.988	96.105	**96.880**
arrhythmia		59.271	64.377	61.512	**66.357**	67.937	67.705	67.266	**70.130**	69.266	68.150	69.053	**71.242**	68.159	**72.353**	72.135	71.469
audiology		51.759	51.759	57.589	**58.379**	65.929	63.775	67.312	**70.810**	65.949	67.292	72.095	**74.783**	70.296	70.771	76.482	**79.644**
autos		47.333	45.405	**54.143**	52.262	53.595	59.405	**66.333**	60.857	66.405	68.214	69.690	**69.762**	68.738	72.643	**75.595**	72.643
balance-scale		83.669	83.513	**85.261**	84.470	84.956	85.750	**86.892**	86.879	87.837	87.046	87.673	**88.966**	88.490	89.601	89.913	**91.027**
breast-cancer		64.335	66.096	**69.224**	67.180	69.286	70.320	69.963	**70.345**	73.042	72.709	73.067	**73.448**	69.224	69.594	69.544	**72.007**
bridges-version1		**50.455**	**50.455**	**50.455**	**50.455**	56.091	53.182	**58.091**	51.091	59.273	**65.636**	60.182	63.818	58.182	64.727	**67.727**	60.091
bridges-version2		**48.455**	**48.455**	**48.455**	**48.455**	54.273	51.273	**62.909**	57.818	54.727	58.273	58.182	**61.818**	57.091	58.364	64.909	**66.818**
clevalend		74.172	77.527	76.892	**79.462**	81.817	79.860	**82.161**	81.151	81.151	81.806	82.129	**82.194**	83.462	82.473	82.817	**84.441**
cmc		49.764	**49.968**	49.492	49.899	49.492	**52.472**	51.048	51.661	51.388	53.764	**54.780**	52.064	52.677	**54.035**	52.882	53.899
column_2C		80.645	80.645	78.710	**82.258**	79.032	80.323	**83.548**	83.226	81.613	80.645	81.613	**82.903**	82.258	82.581	83.226	**84.516**
column_3C		80.000	74.839	80.000	**82.258**	79.032	80.645	81.613	**86.452**	80.323	80.000	81.613	**86.774**	83.871	**84.839**	84.194	83.871
credit-rating		83.478	83.188	84.493	**85.072**	85.072	85.072	84.638	**85.507**	85.507	85.797	**86.522**	86.232	85.072	85.652	**87.101**	86.957
cylinder-bands		60.000	61.667	63.519	**63.889**	64.444	63.519	**69.815**	68.333	70.000	69.259	69.815	**75.000**	70.556	68.148	**76.852**	75.185
dermatology		85.008	84.452	92.342	**95.901**	95.631	94.272	95.368	**97.290**	94.827	94.264	96.186	**98.101**	96.742	96.734	**97.830**	97.553
ecoli		75.303	73.529	75.276	**83.307**	80.633	79.750	83.913	**86.007**	83.913	83.351	85.071	**86.292**	83.601	84.198	85.107	**87.460**
flags		50.132	**52.158**	50.184	51.579	50.658	51.605	**55.289**	53.263	**58.316**	54.789	55.763	57.737	56.316	53.763	57.263	**60.026**
german_credit		68.500	71.500	70.900	**72.300**	73.200	**73.500**	73.200	73.300	73.200	**74.100**	74.000	73.700	72.900	73.900	74.100	**76.000**
glass		51.407	54.610	**57.511**	56.039	63.528	63.528	63.506	**70.563**	61.710	63.203	67.792	**69.675**	64.004	64.978	**70.519**	70.498
haberman		69.258	69.935	**71.860**	71.527	72.505	73.161	71.559	**74.849**	72.860	72.516	**74.516**	73.538	**73.849**	73.839	70.258	72.860
heart-statlog		76.296	72.593	**79.630**	77.407	80.000	78.148	79.630	**80.370**	79.259	80.000	**82.222**	80.741	79.259	78.889	**82.222**	80.741
hepatitis		**82.542**	79.833	80.625	77.917	**82.625**	80.708	82.500	82.583	82.500	**84.500**	80.583	82.542	81.167	82.500	80.667	**87.042**
horse-colic		77.155	77.147	78.544	**79.324**	82.628	**83.431**	82.583	83.393	82.853	83.408	80.435	**83.964**	83.408	84.227	83.979	**85.045**
hungarian-heart		81.310	82.333	**82.667**	82.632	83.345	**83.368**	79.632	80.989	82.034	80.023	81.644	**82.345**	81.310	**82.356**	82.011	81.333
hypothyroid		96.873	97.270	97.615	**99.496**	98.728	98.648	98.728	**99.603**	98.807	98.304	98.781	**99.364**	99.073	98.781	99.126	**99.417**
ionosphere		80.095	82.087	89.468	**90.357**	89.190	90.881	90.889	**94.325**	91.468	92.032	91.484	**94.603**	91.460	92.024	92.897	**94.032**
iris		85.333	85.333	**94.000**	88.000	92.667	93.333	94.000	**94.667**	**96.667**	95.333	94.667	96.000	**97.333**	95.333	96.000	96.000
kr-vs-kp		94.932	94.647	96.840	**98.499**	97.090	96.933	98.154	**99.156**	97.467	97.245	98.717	**99.031**	97.248	97.935	98.593	**99.343**
labor		**72.667**	**72.667**	**72.667**	**72.667**	73.667	66.333	**82.333**	80.667	82.333	77.000	78.333	**85.667**	78.333	78.667	84.333	**90.000**
letter		81.565	81.565	84.680	**88.220**	87.230	87.190	89.825	**93.270**	89.825	89.410	92.015	**94.575**	91.635	90.890	93.400	**95.260**
lymphography		69.619	71.714	71.714	**73.048**	74.905	78.381	74.238	**79.000**	71.619	78.333	**80.333**	77.143	82.381	77.619	81.000	**83.143**
mushroom		99.643	99.643	99.889	**100.000**	99.914	99.914	99.926	**100.000**	99.951	99.926	99.951	**100.000**	99.951	99.938	99.963	**100.000**
optdigits		92.740	92.544	94.644	**95.819**	94.911	95.071	95.463	**97.189**	95.925	95.463	96.495	**97.740**	96.335	96.139	97.153	**97.473**
page-blocks		95.980	95.432	96.072	**97.296**	96.528	96.254	96.766	**97.460**	96.583	96.711	97.040	**97.606**	96.930	97.058	97.205	**97.552**
pendigits		96.889	96.934	97.626	**98.699**	97.771	97.926	98.535	**99.045**	98.590	98.399	98.826	**99.118**	98.754	98.672	98.917	**99.118**
pima_diabetes		72.011	72.927	74.219	**74.352**	74.222	76.300	**77.218**	76.174	76.174	74.614	75.005	**76.304**	75.138	74.610	75.140	**75.781**
postoperative		**64.444**	**64.444**	**64.444**	**64.444**	**70.000**	66.667	64.444	66.667	**70.000**	**70.000**	66.667	68.889	64.444	67.778	66.667	**70.000**
primary-tumor		32.701	30.945	36.881	**38.012**	37.415	38.333	41.854	**42.709**	41.854	40.963	39.528	**42.442**	**43.681**	42.478	43.351	42.166
segment		93.463	93.853	94.545	**95.411**	95.411	94.459	95.887	**97.965**	96.190	96.104	97.186	**97.965**	96.494	96.450	97.143	**98.139**
sick		97.720	97.190	97.905	**98.621**	98.091	98.038	98.144	**98.860**	98.224	97.985	98.330	**98.913**	98.383	98.250	98.701	**99.046**
solar-flare		66.657	68.468	70.017	**71.152**	70.766	70.821	70.541	**71.115**	70.724	70.360	70.817	**72.454**	70.328	71.674	71.551	**73.115**
sonar		62.429	64.952	**67.286**	64.429	67.405	67.286	70.143	**75.429**	73.548	73.571	**79.786**	73.071	76.929	79.286	**81.333**	79.310
soybean		72.594	74.205	81.831	**83.282**	86.087	85.789	89.156	**92.093**	88.574	88.568	92.822	**93.849**	90.774	91.213	93.129	**94.286**
spambase		91.632	91.828	91.958	**93.740**	92.806	93.306	93.414	**95.088**	93.306	93.892	94.088	**95.305**	93.784	93.697	94.762	**95.196**
spect		67.219	67.603	**68.166**	68.137	72.349	72.025	73.351	**82.388**	74.594	73.515	**77.094**	74.402	74.827	**79.224**	77.171	77.828
sponge		**92.500**	**92.500**	**92.500**	**92.500**	**92.500**	**92.500**	**92.500**	**92.500**	**92.500**	**92.500**	**92.500**	**92.500**	91.071	92.500	**93.750**	92.500
tae		33.708	39.042	**44.375**	43.042	41.750	37.708	**46.333**	41.000	44.333	48.333	50.333	**56.333**	47.583	50.250	**56.875**	**56.875**
tic-tac-toe		74.637	74.536	76.724	**80.484**	80.586	81.109	83.813	**89.148**	84.766	83.409	89.038	**94.677**	89.878	88.315	93.940	**96.453**
vehicle		69.979	66.085	71.535	**71.625**	73.889	71.406	75.892	**76.714**	73.641	72.580	74.711	**77.076**	75.416	72.464	75.305	**76.134**
vote		91.723	91.047	93.108	**94.937**	**95.645**	94.271	94.963	95.180	94.244	95.190	95.634	**96.327**	96.327	94.952	**96.559**	**96.559**
vowel		45.152	45.657	56.667	**57.980**	67.374	62.222	74.646	**78.384**	77.374	73.636	84.747	**88.889**	84.141	82.121	91.313	**95.253**
waveform		81.320	81.820	**82.480**	82.200	82.180	83.140	82.700	**83.340**	82.700	**83.820**	83.160	82.940	83.320	**84.080**	83.180	83.880
wine		87.680	86.536	87.157	**96.078**	90.425	92.157	**95.556**	94.967	93.333	93.856	**97.190**	96.634	94.412	96.078	96.078	**96.634**
wisconsin-breast		95.418	95.277	95.994	**97.137**	96.277	96.422	96.565	**96.994**	96.849	96.708	96.708	**96.851**	96.563	96.565	96.708	**97.280**
zoo		**70.455**	**70.455**	**70.455**	**70.455**	81.273	78.273	81.364	**87.273**	83.273	83.273	88.273	**93.091**	88.273	89.273	89.273	**92.182**
**Average**		73.913	74.205	76.356	**77.264**	78.418	78.171	80.169	**81.263**	80.306	80.424	81.636	**82.975**	81.213	81.645	83.163	**83.963**

**Table 4:** Classification accuracies of XGBoost on four different ratios.

		*R* = 10%				*R* = 20%				*R* = 30%				*R* = 40%			
	Method	Supervised	Semi-Supervised	Active Random	Combination	Supervised	Semi-Supervised	Active Random	Combination	Supervised	Semi-Supervised	Active Random	Combination	Supervised	Semi-Supervised	Active Random	Combination
Dataset																	
anneal		92.759	92.648	95.100	**96.994**	96.323	96.101	96.881	**98.885**	96.993	96.881	97.437	**98.884**	97.438	97.105	97.772	**98.884**
arrhythmia		55.531	56.420	**63.092**	61.947	63.285	64.831	65.498	**68.155**	65.498	68.372	68.174	**70.372**	67.493	67.053	70.357	**72.150**
audiology		48.261	45.217	**52.668**	50.119	57.134	58.024	**62.905**	61.107	63.360	65.534	68.103	**69.032**	66.364	67.668	72.964	**76.976**
autos		46.333	44.857	**51.143**	50.738	58.952	55.548	**65.357**	65.310	64.476	64.429	70.643	**77.571**	72.690	72.167	77.952	**78.976**
balance-scale		76.792	75.202	77.747	**79.813**	80.632	80.952	**82.396**	81.126	82.568	82.401	**83.689**	83.039	83.039	83.835	**84.944**	83.041
breast-cancer		65.788	64.704	66.084	**67.488**	67.167	66.810	66.392	**67.857**	65.000	67.475	66.392	**68.140**	66.047	68.842	65.382	**71.613**
bridges-version1		**42.818**	**42.818**	**42.818**	**42.818**	44.818	47.636	**54.455**	49.545	**60.909**	57.182	54.364	59.000	56.364	54.273	57.000	**60.909**
bridges-version2		**43.545**	**43.545**	**43.545**	**43.545**	42.727	43.727	**55.273**	54.091	**60.182**	57.273	56.182	58.182	57.182	56.273	58.000	**67.909**
clevalend		71.172	72.516	**75.849**	73.796	79.548	**80.860**	80.785	80.505	79.484	80.473	**84.430**	80.473	**82.796**	81.140	82.441	81.785
cmc		49.220	49.488	**50.776**	50.576	51.253	52.951	53.358	**54.312**	53.563	54.987	54.442	**55.052**	53.966	54.848	54.304	**54.852**
column_2C		76.129	76.452	76.129	**80.645**	81.613	80.968	80.323	**83.548**	79.677	80.968	81.613	**81.935**	**85.806**	80.000	83.226	83.226
column_3C		**79.032**	77.419	76.129	78.065	81.290	81.290	80.645	**81.613**	80.645	80.323	79.355	**82.581**	80.323	82.903	80.968	**84.839**
credit-rating		82.174	82.029	**83.478**	**83.478**	83.188	84.058	83.913	**85.652**	84.203	84.928	84.638	**86.812**	84.783	85.072	**86.522**	**86.522**
cylinder-bands		65.926	63.333	**70.370**	69.444	71.667	71.667	71.852	**74.630**	75.741	74.815	77.963	**78.148**	75.000	74.444	79.259	**80.000**
dermatology		83.889	85.000	89.872	**92.342**	93.183	92.102	94.820	**96.749**	94.820	94.002	95.375	**96.194**	94.827	94.550	95.375	**97.020**
ecoli		68.146	67.273	70.865	**76.203**	75.000	75.000	81.836	**82.121**	81.836	80.339	81.827	**84.804**	80.321	81.221	82.727	**84.528**
flags		49.132	48.632	48.579	**51.053**	48.632	50.711	52.237	**54.711**	53.237	51.632	**53.632**	49.000	53.658	50.579	**59.737**	56.289
german_credit		68.900	68.800	70.100	**70.700**	72.500	71.500	73.500	**73.800**	73.500	71.900	72.800	**73.900**	73.200	73.800	74.100	**75.100**
glass		46.818	46.364	54.264	**55.130**	62.121	60.714	**64.978**	63.117	64.545	65.952	66.342	**67.424**	67.359	67.338	69.524	**72.381**
haberman		66.591	67.269	69.290	**71.903**	69.247	**69.570**	67.645	68.914	68.290	67.602	67.333	**70.280**	**70.925**	69.935	66.656	67.312
heart-statlog		71.852	72.963	**74.444**	74.074	**75.926**	74.074	75.556	75.185	75.926	76.296	75.926	**80.370**	74.815	74.444	**80.370**	78.519
hepatitis		74.042	73.417	76.000	**77.333**	74.792	75.333	**79.292**	77.333	80.542	**80.583**	77.250	80.000	77.958	78.000	77.208	**81.167**
horse-colic		75.788	75.556	**80.165**	79.092	79.617	80.721	81.809	**82.875**	79.610	80.691	81.239	**84.767**	80.976	80.698	81.246	**85.586**
hungarian-heart		80.989	79.644	79.310	**81.977**	80.667	**81.690**	79.966	78.563	**78.931**	**78.931**	76.862	78.609	79.943	79.230	79.943	**80.264**
hypothyroid		98.304	98.304	98.436	**99.470**	98.781	98.754	99.046	**99.682**	99.046	99.046	99.311	**99.682**	99.284	99.205	99.523	**99.655**
ionosphere		80.659	80.659	84.635	**84.937**	86.643	86.365	88.603	**90.333**	87.183	85.190	88.611	**92.032**	91.175	88.317	90.897	**92.032**
iris		84.667	82.000	86.667	**91.333**	92.000	91.333	**94.667**	93.333	94.667	93.333	94.667	**95.333**	94.667	94.667	**96.000**	**96.000**
kr-vs-kp		96.339	96.151	97.059	**98.561**	97.685	97.434	98.279	**99.437**	98.310	98.216	98.873	**99.500**	98.811	98.780	99.062	**99.343**
labor		**65.000**	**65.000**	**65.000**	**65.000**	64.667	72.000	**73.667**	59.333	73.667	78.667	**79.000**	78.667	79.000	**82.667**	80.333	82.000
letter		82.325	82.095	84.905	**87.820**	86.930	86.430	89.330	**92.050**	89.330	88.795	90.910	**93.235**	90.585	89.990	91.760	**93.425**
lymphography		70.238	69.571	**74.286**	66.952	76.333	75.000	76.286	**77.000**	77.762	76.333	78.333	**82.524**	79.667	77.667	83.095	**85.190**
mushroom		99.729	99.717	99.877	**100.000**	99.914	99.914	99.914	**100.000**	99.914	99.963	99.963	**100.000**	99.951	**100.000**	99.963	**100.000**
optdigits		91.299	90.694	93.345	**95.071**	94.537	94.431	95.196	**97.349**	95.214	95.107	96.068	**97.331**	96.050	95.516	96.370	**97.331**
page-blocks		95.834	95.889	96.199	**97.296**	96.382	96.437	96.602	**97.387**	96.583	96.583	97.186	**97.223**	96.857	96.985	97.241	**97.332**
pendigits		94.642	94.214	90.897	**97.844**	96.761	96.543	97.398	**98.854**	97.380	97.416	97.971	**98.890**	97.726	97.689	98.308	**98.836**
pima_diabetes		72.138	70.976	72.143	**74.096**	74.482	**75.784**	74.229	75.511	**74.626**	73.959	73.717	74.098	74.621	74.489	72.931	**75.665**
postoperative		**60.000**	**60.000**	**60.000**	**60.000**	58.889	60.000	60.000	**67.778**	58.889	58.889	**61.111**	**61.111**	**57.778**	**57.778**	**57.778**	55.556
primary-tumor		34.777	37.736	**40.677**	39.519	38.592	40.963	**43.619**	40.695	43.619	44.795	43.324	**46.310**	42.754	43.048	**44.822**	43.057
segment		92.857	91.948	93.680	**95.844**	94.719	94.156	95.714	**98.052**	95.671	95.584	96.623	**98.398**	96.364	96.190	97.619	**98.312**
sick		97.614	97.640	97.799	**98.860**	98.171	98.250	98.356	**99.046**	98.356	98.330	98.356	**99.046**	98.303	98.250	98.781	**99.072**
solar-flare		68.911	68.786	**70.739**	70.238	69.480	69.089	70.392	**70.956**	70.408	70.345	70.122	**72.730**	70.513	72.118	72.127	**72.945**
sonar		58.143	59.071	**63.833**	62.976	68.714	65.786	**71.619**	69.619	75.405	71.119	**77.333**	74.952	74.929	74.857	77.381	**80.667**
soybean		72.598	72.157	**81.249**	79.776	85.200	84.327	89.009	**90.473**	89.450	87.835	90.190	**93.847**	90.040	90.332	92.971	**93.252**
spambase		91.480	90.936	92.480	**93.827**	93.284	92.610	93.284	**94.870**	93.588	93.306	94.392	**95.153**	94.371	93.870	94.653	**94.957**
spect		62.936	63.129	63.478	**65.166**	66.863	69.601	67.959	**70.157**	70.467	69.464	**73.853**	73.722	70.027	72.094	76.070	**76.400**
sponge		**92.500**	**92.500**	**92.500**	**92.500**	**92.500**	**92.500**	91.071	**92.500**	**93.750**	**93.750**	**93.750**	92.321	**93.750**	92.321	**93.750**	93.571
tae		34.417	31.750	**37.667**	37.083	34.958	39.000	39.083	**48.292**	41.042	40.333	**49.667**	42.292	46.333	46.333	**57.625**	53.000
tic-tac-toe		78.195	78.922	81.427	**87.067**	88.207	86.851	91.132	**95.827**	91.757	91.237	95.095	**97.705**	95.616	94.991	96.346	**98.224**
vehicle		61.941	63.489	68.331	**68.922**	67.265	67.034	69.634	**72.224**	70.339	70.571	**73.646**	73.637	71.408	70.455	74.933	**75.312**
vote		95.185	95.185	95.412	**96.105**	95.640	94.958	**95.877**	95.640	95.185	95.645	**96.094**	95.640	95.407	95.412	**95.645**	95.640
vowel		49.293	50.707	**57.273**	56.667	63.333	62.323	72.222	**74.848**	71.818	70.606	78.283	**82.525**	77.273	75.455	83.939	**88.384**
waveform		81.780	80.480	82.160	**83.160**	82.340	82.220	83.160	**83.780**	83.160	83.440	83.620	**84.380**	84.280	83.960	84.300	**84.580**
wine		83.203	83.758	85.980	**87.647**	92.124	92.092	93.856	**94.967**	93.268	93.824	**94.412**	**94.412**	94.412	93.235	94.935	**96.634**
wisconsin-breast		93.561	94.275	94.135	**96.420**	94.418	94.133	95.137	**95.994**	95.137	94.849	95.280	**95.851**	95.422	94.708	94.994	**95.851**
zoo		**60.545**	**60.545**	**60.545**	**60.545**	79.273	80.273	83.273	**84.091**	87.273	87.273	90.273	**93.091**	90.273	89.273	89.273	**96.000**
**Average**		72.413	72.179	74.557	**75.454**	76.734	76.971	78.896	**79.632**	79.378	79.232	80.474	**81.640**	80.380	80.110	81.844	**83.056**

**Table 5:** Classification accuracies of voting (RF, RotF, XGBoost) on four different ratios.

		*R* = 10%				*R* = 20%				*R* = 30%				*R* = 40%			
	Method	Supervised	Semi-Supervised	Active Random	Combination	Supervised	Semi-Supervised	Active Random	Combination	Supervised	Semi-Supervised	Active Random	Combination	Supervised	Semi-Supervised	Active Random	Combination
Dataset																	
anneal		90.864	89.634	92.869	**94.648**	94.207	94.096	96.768	**98.658**	96.323	96.660	97.552	**99.107**	97.552	97.663	98.218	**98.886**
arrhythmia		60.836	58.865	63.734	**65.729**	67.266	66.169	66.604	**68.362**	67.715	67.271	70.821	**71.705**	69.478	67.261	72.575	**73.681**
audiology		51.759	50.000	**58.419**	57.036	59.842	62.451	**67.273**	66.383	66.818	68.123	72.964	**73.458**	68.577	71.166	76.462	**78.241**
autos		49.262	45.905	51.667	**55.619**	59.905	59.357	**67.810**	65.786	68.357	67.810	74.548	**78.095**	73.143	73.143	78.452	**79.857**
balance-scale		80.947	79.355	81.423	**83.187**	83.034	83.510	**85.289**	84.002	84.808	84.647	**85.123**	84.813	86.078	86.400	**87.355**	87.353
breast-cancer		66.810	68.214	66.798	**69.273**	67.512	67.894	68.165	**72.759**	69.187	69.557	67.475	**71.342**	68.498	68.867	67.118	**73.054**
bridges-version1		**46.545**	**46.545**	**46.545**	**46.545**	53.273	49.455	56.273	**57.182**	62.909	59.909	60.182	**65.727**	60.182	65.727	65.727	**67.545**
bridges-version2		**47.545**	**47.545**	**47.545**	**47.545**	47.545	51.091	**59.818**	54.455	59.182	61.909	62.091	**65.909**	61.091	64.818	**67.545**	63.909
clevalend		72.495	74.828	78.172	**79.817**	82.183	79.871	81.430	**84.140**	81.441	81.785	**83.774**	82.796	83.774	83.441	**84.452**	82.462
cmc		50.376	50.645	51.187	**51.732**	51.731	**53.292**	52.883	52.753	52.475	**55.661**	53.967	54.238	52.679	**55.933**	53.422	53.972
column_2C		78.387	78.065	78.710	**80.000**	80.645	81.290	83.226	**85.484**	82.258	82.258	83.226	**83.548**	85.161	83.226	**86.129**	85.484
column_3C		79.677	78.710	79.032	**82.258**	81.613	81.290	83.871	**86.129**	83.548	82.581	82.258	**83.871**	82.581	83.226	82.258	**83.871**
credit-rating		83.768	84.493	84.493	**85.797**	85.072	83.768	85.362	**86.232**	85.652	85.507	85.942	**86.232**	86.522	86.232	86.812	**87.536**
cylinder-bands		67.407	66.852	**70.556**	70.000	72.222	70.741	**75.556**	73.333	75.926	70.926	78.148	**78.519**	76.481	72.963	81.111	**82.037**
dermatology		86.089	86.622	93.431	**95.616**	95.901	95.623	96.456	**97.020**	96.456	97.005	**97.568**	97.553	96.742	97.275	96.734	**97.568**
ecoli		74.706	73.779	76.488	**80.945**	79.135	78.547	83.021	**84.822**	83.021	81.818	85.704	**85.971**	84.207	83.030	85.722	**87.469**
flags		53.211	47.579	**53.263**	51.053	55.921	52.184	55.316	**56.237**	**59.395**	56.289	56.289	57.789	55.237	56.789	**60.816**	58.921
german_credit		70.900	70.700	71.600	**73.900**	74.300	74.200	74.800	**75.500**	74.800	**75.400**	74.900	74.800	75.700	73.900	75.500	**76.300**
glass		54.719	55.173	60.390	**61.710**	66.364	65.909	67.792	**69.221**	66.840	68.247	71.970	**74.762**	70.519	70.022	75.173	**76.212**
haberman		70.**591**	70.258	67.624	69.925	70.237	70.559	69.280	**73.151**	70.581	68.946	**71.570**	71.237	71.269	70.914	70.892	70.570
heart-statlog		72.963	74.074	76.296	**77.778**	78.889	76.667	77.778	**80.741**	77.037	77.778	79.630	**82.222**	79.630	79.259	**82.963**	81.111
hepatitis		79.250	81.833	76.708	**81.875**	78.792	80.667	80.000	**83.792**	81.875	81.875	79.875	**82.500**	77.958	81.833	80.500	**82.542**
horse-colic		77.995	78.799	81.802	**82.883**	81.794	83.956	83.694	**84.505**	83.138	83.949	82.868	**86.396**	84.767	84.227	84.234	**86.404**
hungarian-heart		80.655	80.621	80.977	**83.391**	82.690	**83.345**	80.632	81.678	82.000	**82.356**	78.897	79.966	80.632	80.966	**82.333**	80.644
hypothyroid		98.013	97.827	98.463	**99.709**	98.940	98.781	99.099	**99.709**	99.046	99.125	99.284	**99.735**	99.231	99.258	99.497	**99.655**
ionosphere		81.516	80.944	88.063	**91.206**	90.341	89.778	90.317	**93.175**	90.325	91.175	**92.603**	**92.603**	91.460	92.889	92.889	**93.746**
iris		82.000	82.667	91.333	**92.667**	92.667	92.667	94.667	**95.333**	94.667	94.667	**95.333**	94.667	94.667	94.667	**95.333**	**95.333**
kr-vs-kp		96.402	96.308	97.121	**98.686**	97.747	97.403	98.279	**99.468**	98.435	98.310	98.967	**99.374**	98.842	98.779	99.124	**99.437**
labor		**68.000**	**68.000**	**68.000**	**68.000**	75.333	73.667	**78.667**	74.667	78.667	78.667	80.333	**80.667**	80.333	79.000	82.667	**84.667**
letter		85.625	85.410	88.460	**91.850**	90.355	89.950	92.360	**95.315**	92.360	91.950	93.955	**96.255**	93.450	93.225	94.875	**96.335**
lymphography		71.000	71.714	**75.667**	69.714	76.905	**80.333**	77.667	77.524	76.381	76.286	81.000	**81.714**	80.381	80.333	**83.762**	83.667
mushroom		99.729	99.729	99.914	**100.000**	99.914	99.926	99.926	**100.000**	99.951	99.926	99.975	**100.000**	99.963	99.963	99.975	**100.000**
optdigits		94.217	93.968	95.534	**97.384**	96.246	96.157	96.922	**98.238**	97.064	96.779	97.456	**98.274**	97.171	97.046	97.562	**98.025**
page-blocks		95.980	96.071	96.346	**97.643**	96.602	96.620	96.784	**97.570**	96.857	96.839	97.333	**97.424**	97.077	97.186	97.479	**97.607**
pendigits		97.034	97.052	97.498	**98.717**	98.008	97.999	98.544	**99.327**	98.408	98.581	98.790	**99.300**	98.699	98.581	98.999	**99.263**
pima_diabetes		72.925	74.238	74.231	**76.176**	75.137	76.049	**76.316**	75.658	**77.093**	75.531	75.540	75.911	75.398	74.879	74.626	**75.660**
postoperative		**67.778**	**67.778**	**67.778**	**67.778**	64.444	65.556	64.444	**68.889**	63.333	**65.556**	63.333	**65.556**	62.222	**64.444**	63.333	**64.444**
primary-tumor		35.365	36.257	**37.727**	36.533	39.492	40.695	**46.586**	43.922	**46.586**	45.089	43.039	45.989	44.840	42.763	**45.989**	44.831
segment		93.853	93.853	94.935	**96.667**	95.498	95.844	96.104	**98.485**	96.277	96.320	97.186	**98.485**	96.840	96.883	97.835	**98.528**
sick		97.826	97.720	97.985	**98.595**	98.197	98.303	98.356	**99.072**	98.356	98.276	98.409	**99.019**	98.383	98.409	98.807	**99.019**
solar-flare		69.171	68.081	70.975	**71.101**	70.275	70.458	70.643	**72.026**	70.477	70.524	70.394	**72.741**	70.805	70.931	70.971	**72.838**
sonar		62.452	61.452	**66.786**	66.357	70.190	68.286	74.024	**75.500**	73.524	74.452	**79.262**	76.905	78.786	76.881	78.857	**81.214**
soybean		75.816	74.938	82.711	**85.049**	85.931	86.228	90.030	**92.530**	90.471	89.595	92.234	**94.290**	91.211	92.238	94.150	**94.578**
spambase		92.349	92.088	93.241	**94.370**	93.654	93.545	94.045	**95.349**	94.175	94.088	94.631	**95.544**	94.479	94.284	94.979	**95.327**
spect		63.985	64.911	65.652	**66.592**	**73.421**	71.902	69.779	72.203	73.337	72.457	**77.842**	74.455	75.135	76.763	**81.068**	77.279
sponge		**92.500**	**92.500**	**92.500**	**92.500**	**92.500**	**92.500**	**92.500**	**92.500**	**92.500**	**92.500**	**92.500**	**92.500**	92.500	92.500	**93.750**	92.500
tae		35.708	37.750	**43.708**	41.708	38.958	37.000	41.667	**43.583**	42.333	41.083	47.667	**51.708**	51.000	49.000	**56.917**	56.333
tic-tac-toe		78.502	78.712	80.907	**86.634**	87.477	86.435	90.919	**96.036**	90.611	88.732	94.781	**98.330**	95.198	92.177	97.598	**98.641**
vehicle		66.203	66.326	**71.175**	69.273	71.052	70.920	72.944	**75.661**	72.825	69.864	**75.190**	73.529	72.469	71.164	76.361	**76.480**
vote		94.958	94.271	95.180	**96.554**	95.185	95.418	96.099	**96.327**	95.872	96.094	**96.554**	96.099	95.867	**96.321**	96.099	95.872
vowel		49.697	49.798	**60.707**	59.091	68.485	65.051	77.980	**80.101**	77.374	75.960	86.162	**91.212**	84.949	83.838	92.222	**95.758**
waveform		83.580	83.320	83.980	**84.620**	84.180	84.100	84.640	**84.880**	84.640	84.420	**85.400**	84.960	85.140	85.100	**85.380**	85.220
wine		86.569	84.869	85.458	**96.667**	93.791	95.458	96.111	**98.333**	96.111	94.967	96.634	**97.745**	96.667	96.634	96.634	**97.745**
wisconsin-breast		94.849	94.563	95.422	**96.708**	95.708	95.275	95.994	**96.280**	95.849	95.708	95.994	**96.280**	96.137	95.565	**96.280**	**96.280**
zoo		**75.273**	**75.273**	**75.273**	**75.273**	83.273	82.273	82.273	**91.182**	87.273	86.273	88.273	**95.091**	88.273	88.273	89.273	**94.273**
**Average**		74.666	74.500	76.772	**78.038**	78.909	78.737	80.614	**81.839**	80.962	80.692	82.244	**83.435**	81.928	81.968	83.742	**84.294**

**Table 6:** Friedman aligned ranking test and Holm’s post hoc test regarding BagDT (*a = 0.10*).

Labeled Ratio (R)	Classifier (BagDT)	Friedman p-Value (Statistic)	Friedman Ranking	Holm’s Post Hoc Test	
				p-Value	Null Hypothesis
**10%**	**Combination**	**0.00000** **(76.02618)**	55.83636	-	-
	Active Random		81.33636	0.03566	rejected
	Supervised		148.77273	0.00000	rejected
	Semi-supervised		156.05455	0.00000	rejected
20%	Combination	0.00000 (59.02259)	57.93636	-	-
	Active Random		91.92727	0.00510	rejected
	Supervised		141.07273	0.00000	rejected
	Semi-supervised		151.06364	0.00000	rejected
30%	Combination	0.00000 (78.32732)	48.57273	-	-
	Active Random		91.35455	0.00042	rejected
	Supervised		148.44545	0.00000	rejected
	Semi-supervised		153.62727	0.00000	rejected
40%	Combination	0.00000 (65.36953)	61.73636	-	-
	Active Random		85.82727	0.04717	rejected
	Supervised		129.42727	0.00000	rejected
	Semi-supervised		165.00909	0.00000	rejected

**Table 7:** Friedman aligned ranking test and Holm’s post hoc test regarding RF (*a = 0.10*).

Labeled Ratio (R)	Classifier (Random Forests)	Friedman p-Value (Statistic)	Friedman Ranking	Holm’s Post Hoc Test	
				p-Value	Null Hypothesis
**10** **%**	**Combination**	**0.00000** **(90.74521)**	50.23636	-	-
	Active Random		80.05455	0.01403	rejected
	Supervised		153.30000	0.00000	rejected
	Semi-supervised		158.40909	0.00000	rejected
20%	Combination	0.00000 (76.85983)	52.23636	-	-
	Active Random		88.34545	0.00293	rejected
	Supervised		142.39091	0.00000	rejected
	Semi-supervised		159.02727	0.00000	rejected
30%	Combination	0.00000 (76.55845)	55.79091	-	-
	Active Random		83.12727	0.02432	rejected
	Supervised		140.62727	0.00000	rejected
	Semi-supervised		162.45455	0.00000	rejected
40%	Combination	0.00000 (59.66724)	61.71818	-	-
	Active Random		90.20000	0.01895	rejected
	Supervised		129.20000	0.00000	rejected
	Semi-supervised		160.88182	0.00000	rejected

**Table 8:** Friedman aligned ranking test and Holm’s post hoc test regarding RotF (*a = 0.10*).

Labeled Ratio (R)	Classifier (Rotation Forest)	Friedman p-Value (Statistic)	Friedman Ranking	Holm’s Post Hoc Test	
				p-Value	Null Hypothesis
**10%**	**Combination**	**0.00000** **(86.92304)**	53.12727	-	-
	Active Random		78.83636	0.03417	rejected
	Semi-supervised		149.38182	0.00000	rejected
	Supervised		160.65455	0.00000	rejected
20%	Combination	0.00000 (68.42331)	53.53636	-	-
	Active Random		91.30909	0.00186	rejected
	Supervised		148.11818	0.00000	rejected
	Semi-supervised		149.03636	0.00000	rejected
30%	Combination	0.00000 (61.06200)	54.55455	-	-
	Active Random		95.74545	0.00069	rejected
	Semi-supervised		145.80909	0.00000	rejected
	Supervised		145.89091	0.00000	rejected
40%	Combination	0.00000 (71.23507)	56.67273	-	-
	Active Random		84.93636	0.01989	rejected
	Semi-supervised		141.01818	0.00000	rejected
	Supervised		159.37273	0.00000	rejected

**Table 9:** Friedman aligned ranking test and Holm’s post hoc test regarding extreme gradient boosted trees (XGBoost) (*a = 0.10*).

Labeled Ratio (R)	Classifier (XGBoost)	Friedman p-Value (Statistic)	Friedman Ranking	Holm’s Post Hoc Test	
				p-Value	Null Hypothesis
**10%**	**Combination**	**0.00000** **(100.32586)**	48.66364	-	-
	Active Random		75.80000	0.02538	rejected
	Supervised		153.99091	0.00000	rejected
	Semi-supervised		163.54545	0.00000	rejected
20%	Combination	0.00000 (79.07341)	53.10000	-	-
	Active Random		83.52727	0.01218	rejected
	Semi-supervised		149.70000	0.00000	rejected
	Supervised		155.67273	0.00000	rejected
30%	Combination	0.00000 (64.21611)	53.01818	-	-
	Active Random		95.96364	0.00040	rejected
	Supervised		144.96364	0.00000	rejected
	Semi-supervised		148.05455	0.00000	rejected
40%	Combination	0.00000 (73.61879)	52.05455	-	-
	Active Random		89.32727	0.00214	rejected
	Supervised		147.56364	0.00000	rejected
	Semi-supervised		153.05455	0.00000	rejected

**Table 10:** Friedman aligned ranking test and Holm’s post hoc test regarding voting (RF, RotF, XGBoost) (*a = 0.10*).

Labeled Ratio (R)	Classifier (Voting (RF,RotF,XGBoost))	Friedman p-Value (Statistic)	Friedman Ranking	Holm’s Post Hoc Test	
				p-Value	Null Hypothesis
**10%**	**Combination**	**0.00000** **(94.26061)**	47.87273	-	-
	Active Random		80.97273	0.00639	rejected
	Supervised		155.69091	0.00000	rejected
	Semi-supervised		157.46364	0.00000	rejected
20%	Combination	0.00000 (84.82332)	47.57273	-	-
	Active Random		87.92727	0.00089	rejected
	Supervised		151.62727	0.00000	rejected
	Semi-supervised		154.87273	0.00000	rejected
30%	Combination	0.00000 (71.00226)	53.01818	-	-
	Active Random		89.65455	0.00254	rejected
	Supervised		145.72727	0.00000	rejected
	Semi-supervised		153.60000	0.00000	rejected
40%	Combination	0.00000 (76.77322)	58.08182	-	-
	Active Random		78.40909	0.09400	rejected
	Semi-supervised		150.10909	0.00000	rejected
	Supervised		155.40000	0.00000	rejected

**Table 11:** Friedman aligned ranking test and Holm’s post hoc test regarding k-nearest neighbors (5NN) (*a = 0.10*).

Labeled Ratio (R)	Classifier (5NN)	Friedman p-Value (Statistic)	Friedman Ranking	Holm’s Post Hoc Test	
				*p*-value	Null Hypothesis
**10%**	**Combination**	**0.00000** **(71.86930** **)**	58.98182	-	-
	Active Random		81.24545	0.06662	rejected
	Supervised		141.71818	0.00000	rejected
	Semi-supervised		160.05455	0.00000	rejected
20%	Combination	0.00000 (73.09232)	56.09091	-	-
	Active Random		84.64545	0.01865	rejected
	Supervised		140.45455	0.00000	rejected
	Semi-supervised		160.80909	0.00000	rejected
30%	Combination	0.00000 (62.25286)	57.79091	-	-
	Active Random		89.60000	0.00878	rejected
	Supervised		143.74545	0.00000	rejected
	Semi-supervised		150.86364	0.00000	rejected
40%	Combination	0.00000 (74.33081)	57.08182	-	-
	Active Random		81.72727	0.04231	rejected
	Supervised		144.50909	0.00000	rejected
	Semi-supervised		158.68182	0.00000	rejected

**Table 12:** Friedman aligned ranking test and Holm’s post hoc test regarding logistic (*a = 0.10*).

Labeled Ratio (R)	Classifier (Logistic)	Friedman p-Value (Statistic)	Friedman Ranking	Holm’s Post Hoc Test	
				p-Value	Null Hypothesis
**10%**	**Combination**	**0.00000** **(49.05320)**	64.44545	-	-
	Active Random		90.40000	0.03249	rejected
	Semi-supervised		142.15455	0.00000	rejected
	Supervised		145.00000	0.00000	rejected
20%	Combination	0.00000 (59.73571)	58.75455	-	-
	Active Random		90.32727	0.00929	rejected
	Semi-supervised		143.43636	0.00000	rejected
	Supervised		149.48182	0.00000	rejected
30%	Combination	0.00000 (40.03025)	71.25455	-	-
	Active Random		89.02727	0.14314	accepted
	Supervised		139.65455	0.00000	rejected
	Semi-supervised		142.06364	0.00000	rejected
40%	Combination	0.00000 (65.16112)	61.48182	-	-
	Active Random		81.70909	0.09563	rejected
	Supervised		146.31818	0.00000	rejected
	Semi-supervised		152.49091	0.00000	rejected

**Table 13:** Friedman aligned ranking Test and Holm’s post hoc test regarding logistic model tree (LMT) (*a = 0.10*).

Labeled Ratio (R)	Classifier (LMT)	Friedman p-Value (Statistic)	Friedman Ranking	Holm’s Post Hoc Test	
				p-Value	Null Hypothesis
**10%**	**Combination**	**0.00000** **(74.72391)**	55.78182	-	-
	Active Random		82.53636	0.02751	rejected
	Supervised		150.05455	0.00000	rejected
	Semi-supervised		153.62727	0.00000	rejected
20%	Combination	0.00000 (76.73213)	59.54545	-	-
	Active Random		76.80909	0.15495	accepted
	Semi-supervised		148.55455	0.00000	rejected
	Supervised		157.09091	0.00000	rejected
30%	Combination	0.00000 (50.01495)	56.80000	-	-
	Active Random		103.50909	0.00012	rejected
	Semi-Supervised		139.15455	0.00000	rejected
	Supervised		142.53636	0.00000	rejected
40%	Combination	0.00000 (79.76665)	56.98182	-	-
	Active Random		77.71818	0.08757	rejected
	Semi-Supervised		147.13636	0.00000	rejected
	Supervised		160.16364	0.00000	rejected

**Table 14:** Friedman aligned ranking test and Holm’s post hoc test regarding LogitBoost (*a = 0.10*).

Labeled Ratio (R)	Classifier (LogitBoost)	Friedman p-Value (Statistic)	Friedman Ranking	Holm’s Post Hoc Test	
				p-Value	Null Hypothesis
**10%**	**Combination**	**0.00000** **(75.28847** **)**	52.08182	-	-
	Active Random		87.89091	0.00318	rejected
	Semi-supervised		149.69091	0.00000	rejected
	Supervised		152.33636	0.00000	rejected
20%	Combination	0.00000 (68.38871)	60.38182	-	-
	Active Random		80.80909	0.09239	rejected
	Supervised		147.73636	0.00000	rejected
	Semi-supervised		153.07273	0.00000	rejected
30%	Combination	0.00000 (60.68458)	53.25455	-	-
	Active Random		99.91818	0.00012	rejected
	Supervised		136.14545	0.00000	rejected
	Semi-supervised		152.68182	0.00000	rejected
40%	Combination	0.00000 (46.30018)	70.32727	-	-
	Active Random		86.01818	0.19611	accepted
	Supervised		138.06364	0.00000	rejected
	Semi-supervised		147.59091	0.00000	rejected

## Supplementary Materials

The following are available online at https://www.mdpi.com/1099-4300/21/10/988/s1, Table S1: Classification accuracies of 5 nearest neighbors (5NN) on four different ratios, Table S2: Classification accuracies of logistic regression (logistic) on four different ratios, Table S3: Classification accuracies of logistic model trees (LMT) on four different ratios, Table S4: Classification accuracies of LogitBoost on four different ratios.

## Appendix A

The proposed combination algorithm was implemented as a separate Java package for the WEKA [67] software tool. The decision to develop the combination scheme as a part of the WEKA tool was made since it is one of the most well-known tools used in the machine-learning community, which includes a big number of base learner models. Moreover, it can be easily deployed without requiring programming experience for the end-user. The package can be downloaded using the following link: http://ml.upatras.gr/combine-classification/.
