# Supplementary material for: Combination of Active Learning and Semi-Supervised Learning under a Self-Training Scheme
Source: Entropy (Basel). 2019 Oct 10;21(10):988. doi: 10.3390/e21100988 (PMC7514320; doi:10.3390/e21100988)
Supplement: Supplementary file 1 [file entropy-21-00988-s001.pdf]

Table S1. Classification accuracies of 5 - Nearest Neighbors (5NN) on four different *Ratios.*

Dataset \ Method	*R* = 10%				*R* = 20%				*R* = 30%				*R* = 40%			
	Supervised	Semi-Supervised	Active Random	Combination	Supervised	Semi-Supervised	Active Random	Combination	Supervised	Semi-Supervised	Active Random	Combination	Supervised	Semi-Supervised	Active Random	Combination
anneal	83.739	81.960	85.416	**87.421**	86.195	85.861	87.865	**91.424**	87.865	86.307	88.085	**93.206**	87.975	87.310	90.648	**93.206**
arrhythmia	55.527	54.870	55.092	**55.768**	55.314	54.208	54.865	**55.971**	54.865	53.986	56.411	**57.077**	55.527	54.425	57.285	**58.169**
audiology	35.316	26.542	**43.340**	34.901	47.411	35.810	**52.727**	43.696	53.182	39.427	**55.771**	50.810	**54.012**	43.340	53.933	50.810
autos	39.905	37.000	**41.929**	39.952	41.857	42.786	48.786	**52.571**	49.762	47.214	49.881	**50.690**	50.786	53.095	**55.095**	55.048
balance-scale	82.230	82.063	84.155	**84.790**	84.316	**87.517**	85.755	85.906	85.914	**87.842**	87.040	86.229	87.197	**88.641**	88.152	86.086
breast-cancer	69.951	71.059	69.951	**73.498**	70.702	**71.736**	69.963	71.712	70.308	72.401	71.687	**72.746**	71.700	72.044	70.640	**73.424**
bridges-version1	**43.545**	**43.545**	**43.545**	**43.545**	51.273	**53.273**	52.273	47.455	54.364	55.455	**56.000**	52.455	56.000	**58.091**	57.000	56.273
bridges-version2	**43.727**	**43.727**	**43.727**	**43.727**	47.455	46.545	**53.273**	52.455	52.455	52.364	55.182	**57.182**	56.182	56.091	**57.909**	57.182
clevalend	79.871	**81.505**	77.860	80.161	80.172	80.806	80.473	**83.151**	80.462	81.140	82.796	**84.462**	83.129	81.796	83.118	**83.140**
cmc	42.565	40.735	**44.599**	43.724	43.998	44.265	**44.670**	44.129	44.806	**46.227**	45.686	45.214	45.210	46.975	46.437	**48.611**
column_2C	74.839	74.194	77.097	**79.355**	**79.355**	76.452	78.387	78.065	77.419	77.742	**78.387**	77.742	77.742	78.065	79.032	**81.613**
column_3C	67.097	66.774	69.355	**69.677**	74.194	71.613	**76.129**	75.484	**76.452**	74.516	75.161	74.516	76.129	72.581	75.161	**76.452**
credit-rating	83.478	82.464	84.348	**84.783**	**84.928**	**84.928**	84.348	84.638	84.493	85.217	84.638	**85.942**	85.217	85.507	85.507	**86.377**
cylinder-bands	58.333	58.519	**61.296**	60.556	62.037	60.000	63.704	**64.815**	63.889	64.444	65.556	**66.111**	65.556	64.815	**67.037**	66.296
dermatology	78.941	79.512	85.233	**89.880**	89.077	88.228	92.350	**95.908**	92.905	92.635	94.279	**95.383**	93.731	93.994	94.002	**95.923**
ecoli	71.471	72.059	74.991	**81.560**	80.330	79.750	80.330	**84.804**	80.330	80.339	**84.510**	84.216	83.904	83.619	84.225	**85.686**
flags	**49.079**	43.289	48.974	43.842	44.395	41.842	**50.105**	48.500	50.658	46.921	51.632	**56.737**	52.237	50.079	**59.368**	54.632
german_credit	69.300	69.800	69.400	**71.100**	68.900	68.900	69.800	**71.900**	69.800	70.500	70.700	**70.800**	70.300	69.900	71.700	**72.100**
glass	44.870	43.550	45.866	**47.186**	56.515	54.719	58.442	**62.186**	61.775	60.325	62.208	**64.545**	63.160	60.801	**63.658**	63.593
haberman	70.312	70.290	68.978	**70.989**	72.226	71.258	71.237	**72.559**	71.570	70.591	**73.527**	72.559	71.237	70.280	70.903	**71.581**
heart-statlog	**78.889**	77.778	76.296	76.667	79.630	76.667	76.296	**81.111**	76.296	76.667	**80.000**	79.630	79.259	78.519	**80.741**	78.889
hepatitis	**81.875**	**81.875**	80.042	80.750	79.458	79.458	**83.958**	82.667	83.292	81.375	81.417	**83.792**	82.708	80.083	**83.250**	83.208
horse-colic	**82.853**	77.417	80.143	79.332	**80.428**	80.143	79.887	80.150	79.887	**80.706**	79.887	80.413	80.706	80.983	80.961	**82.057**
hungarian-heart	81.690	82.701	82.713	**83.011**	83.034	**84.057**	83.046	83.368	83.046	**84.402**	81.333	82.000	82.356	**83.011**	82.333	81.989
hypothyroid	92.126	92.100	92.047	**92.710**	92.232	92.445	92.577	**92.975**	92.577	92.657	93.028	**93.266**	92.710	92.710	93.240	**93.266**
ionosphere	70.389	67.817	75.817	**81.532**	78.103	73.817	77.246	**82.095**	76.675	76.103	80.952	**84.040**	80.103	78.103	82.365	**83.754**
iris	71.333	72.000	85.333	**90.000**	89.333	89.333	96.000	**98.000**	96.667	96.667	96.667	**97.333**	**97.333**	96.000	96.667	96.000
kr-vs-kp	81.947	77.222	85.296	**88.268**	86.485	83.761	90.207	**93.963**	90.269	87.734	92.678	**96.465**	92.584	89.079	94.087	**96.872**
labor	**56.000**	**56.000**	**56.000**	**56.000**	71.333	65.000	73.667	**80.667**	73.667	70.000	**79.333**	79.000	79.333	81.000	84.667	**88.000**
letter	77.415	76.345	82.525	**84.095**	85.835	85.535	89.385	**92.310**	89.385	89.545	91.905	**94.970**	91.175	91.710	93.375	**95.425**
lymphography	63.524	62.143	**71.667**	66.238	79.048	72.857	78.286	**83.667**	77.571	78.905	78.238	**81.000**	79.619	77.571	83.000	**83.714**
mushroom	99.335	99.360	99.532	**100.000**	99.791	99.791	99.877	**100.000**	99.877	99.889	99.914	**100.000**	99.889	99.865	99.926	**100.000**
optdigits	95.605	96.299	96.655	**98.292**	97.278	97.598	97.776	**98.665**	97.776	98.043	98.149	**98.541**	98.132	98.185	98.399	**98.541**
page-blocks	93.477	93.514	94.099	**95.487**	94.427	94.336	94.756	**95.652**	94.756	94.573	94.957	**95.798**	94.738	94.829	95.231	**95.725**
pendigits	96.443	96.570	97.207	**99.081**	97.853	98.181	98.499	**99.345**	98.499	98.626	98.872	**99.345**	98.772	98.926	99.127	**99.290**
pima_diabetes	68.882	67.838	69.911	**71.615**	71.350	**71.620**	71.494	71.615	72.013	**72.404**	70.448	71.224	72.273	70.841	71.092	**72.397**
postoperative	**68.889**	**68.889**	**68.889**	**68.889**	68.889	70.000	70.000	**71.111**	**70.000**	**70.000**	68.889	**70.000**	68.889	**71.111**	68.889	**71.111**
primary-tumor	30.045	25.339	34.180	**34.189**	35.945	33.316	**40.989**	37.424	40.989	36.560	**41.872**	39.804	42.460	40.080	**47.175**	45.116
segment	86.926	86.580	89.567	**90.433**	90.606	91.602	92.771	**94.502**	92.727	92.727	93.853	**95.152**	93.377	93.203	94.459	**95.065**
sick	94.379	94.512	94.644	**95.281**	94.988	94.856	95.333	**95.970**	95.333	95.068	95.068	**95.997**	95.069	95.069	95.599	**96.182**
solar-flare	59.885	57.840	**62.572**	61.871	66.335	61.698	**69.347**	67.588	70.133	65.543	**70.174**	68.968	**70.310**	67.553	68.901	70.041
sonar	60.619	57.738	60.190	**60.690**	63.476	63.071	64.952	**71.167**	67.810	70.286	70.262	**73.119**	67.286	71.262	73.619	**77.905**
soybean	53.853	51.081	62.801	**68.201**	71.289	67.624	77.148	**81.982**	77.295	75.107	82.266	**85.784**	81.978	80.215	85.350	**89.738**
spambase	84.937	83.133	85.046	**86.916**	86.241	84.959	87.111	**88.741**	87.067	86.394	87.633	**89.480**	87.937	87.394	88.763	**89.915**
spect	63.953	64.316	66.140	**66.339**	**70.962**	66.524	69.596	64.309	70.105	64.927	69.557	**70.304**	69.557	66.617	69.309	**70.381**
sponge	**92.500**	**92.500**	**92.500**	**92.500**	**92.500**	**92.500**	**92.500**	**92.500**	**92.500**	**92.500**	**92.500**	**92.500**	**92.500**	**92.500**	**92.500**	**92.500**
tae	38.375	39.667	**41.000**	38.958	35.042	34.333	40.375	**42.333**	41.708	42.375	41.708	**47.583**	43.708	49.667	47.667	**51.625**
tic-tac-toe	69.514	70.874	74.745	**76.932**	78.083	75.993	85.390	**91.124**	85.390	82.770	93.633	**98.225**	91.752	85.798	97.807	**98.851**
vehicle	52.482	52.483	56.994	**57.329**	62.920	61.136	**64.200**	63.018	64.436	63.616	66.202	**68.339**	64.667	65.738	67.615	**69.627**
vote	88.531	89.450	89.915	**93.340**	91.744	90.814	91.956	**94.265**	91.956	90.576	91.734	**93.811**	91.728	90.581	92.421	**92.664**
vowel	14.646	11.717	15.051	**16.465**	19.394	17.778	28.687	**29.899**	29.091	26.566	43.838	**47.778**	40.808	37.071	63.939	**65.960**
waveform	76.660	77.460	77.380	**78.440**	78.340	78.720	78.320	**79.060**	78.320	**79.880**	78.220	79.040	78.220	**79.180**	78.960	78.840
wine	79.967	79.902	87.157	**93.268**	94.902	94.314	95.490	**96.667**	95.490	96.667	96.111	**97.190**	96.111	95.556	**96.667**	96.601
wisconsin-breast	95.851	95.851	95.565	**96.565**	96.137	95.994	**96.422**	**96.422**	96.422	95.994	**96.708**	96.565	96.565	95.851	**96.851**	96.565
zoo	**50.636**	**50.636**	**50.636**	**50.636**	69.364	68.364	75.364	**85.182**	80.364	77.364	84.273	**92.182**	84.273	81.273	86.273	**94.091**
**Average**	69.064	68.189	71.007	**71.941**	73.699	72.522	75.534	**76.852**	75.867	75.069	77.298	**78.496**	77.197	76.520	78.946	**79.784**

Table S2. Classification accuracies of Logistic Regression (Logistic) on four different *Ratios*.

Dataset \ Method	***R*** **= 10%** Supervised	Semi-Supervised	Active Random	Combination	***R*** **= 20%** Supervised	Semi-Supervised	Active Random	Combination	***R*** **= 30%** Supervised	Semi-Supervised	Active Random	Combination	***R*** **= 40%** Supervised	Semi-Supervised	Active Random	Combination
anneal	75.943	76.833	**80.620**	78.622	79.844	84.181	83.851	**85.290**	84.072	**86.077**	86.074	85.412	85.628	**88.409**	86.192	86.300
arrhythmia	49.111	46.720	52.000	**54.246**	52.652	54.425	53.546	**55.092**	54.435	52.435	**57.087**	56.203	**55.739**	55.734	54.425	52.213
audiology	36.798	38.439	**45.593**	42.490	49.170	52.668	57.984	**58.419**	57.984	57.075	66.403	**72.095**	65.040	63.300	73.004	**75.632**
autos	41.310	42.405	45.786	**47.714**	52.643	50.190	**56.071**	55.500	57.024	54.595	56.881	**57.548**	56.452	54.571	**64.262**	63.238
balance-scale	85.745	86.723	**89.273**	88.643	89.593	89.593	88.482	**90.714**	88.482	88.641	90.077	**91.672**	88.802	88.802	89.598	**90.230**
breast-cancer	59.113	56.638	53.818	**61.133**	61.884	57.709	**63.202**	60.825	62.143	63.190	**67.833**	66.379	67.069	65.665	68.867	**70.936**
bridges-version1	**46.545**	**46.545**	**46.545**	**46.545**	46.273	52.182	51.455	**53.364**	**56.364**	50.818	55.273	51.273	54.273	51.364	60.364	**62.000**
bridges-version2	**44.545**	**44.545**	**44.545**	**44.545**	50.091	**53.182**	51.091	48.545	55.273	55.273	**56.364**	53.455	55.364	52.727	**60.182**	55.273
clevalend	70.269	71.258	66.591	**72.892**	75.570	76.871	76.570	**77.505**	77.892	77.570	**81.849**	77.871	80.183	79.527	**84.817**	82.183
cmc	45.896	45.623	**49.150**	47.591	49.356	48.474	**51.526**	51.325	51.458	49.352	**51.936**	51.189	51.527	51.121	50.442	**52.274**
column_2C	80.968	80.645	81.613	**83.871**	83.226	83.226	84.516	**84.839**	84.516	84.516	**84.839**	84.194	84.839	**85.161**	84.839	84.516
column_3C	77.097	78.065	77.742	**83.548**	79.032	79.677	**85.161**	84.516	**85.161**	84.516	84.194	84.194	85.161	84.839	**85.806**	85.161
credit-rating	72.464	73.768	72.319	**75.797**	79.275	79.565	**81.884**	81.739	81.304	81.014	**83.333**	83.043	82.899	83.188	**84.203**	**84.203**
cylinder-bands	63.704	61.852	61.667	**66.111**	67.037	68.704	70.000	**71.111**	70.741	66.852	**72.593**	**72.593**	72.407	68.704	72.778	**74.259**
dermatology	87.680	87.695	92.350	**92.635**	93.686	94.009	94.812	**95.083**	95.653	**96.464**	94.557	95.916	95.631	95.893	95.353	**95.916**
ecoli	66.684	70.553	**75.018**	70.597	78.271	77.665	79.162	**80.062**	79.162	78.289	81.845	**83.316**	79.750	80.936	82.415	**84.519**
flags	43.368	44.868	41.368	**45.947**	45.474	44.447	**47.684**	44.816	45.026	45.526	46.000	**50.132**	43.947	40.474	**47.079**	42.395
german_credit	63.400	65.200	**66.400**	66.200	68.200	68.400	**72.400**	70.900	72.400	72.200	**74.500**	73.900	74.300	73.600	**76.300**	75.500
glass	51.905	52.835	54.264	**57.965**	54.177	52.294	**60.238**	58.377	60.693	61.212	60.238	**62.641**	60.671	59.242	**63.009**	61.645
haberman	61.398	61.075	**70.570**	65.634	71.849	71.849	70.903	**74.172**	71.559	70.925	**74.183**	72.839	74.183	73.849	74.806	**75.774**
heart-statlog	68.889	**71.852**	70.370	71.111	75.185	76.667	**80.000**	78.148	80.370	80.000	**81.481**	78.889	80.000	80.000	**82.222**	81.111
hepatitis	76.000	75.458	80.583	**81.292**	74.250	74.208	74.208	**74.417**	**78.083**	77.500	**78.083**	74.917	74.958	71.167	**79.292**	78.625
horse-colic	**70.450**	67.147	69.032	69.362	63.296	66.839	71.456	**73.919**	69.039	68.506	**72.297**	70.646	70.375	72.815	**75.000**	74.452
hungarian-heart	74.839	74.851	**77.598**	74.207	75.885	76.241	**79.655**	78.632	80.322	78.977	**81.000**	77.287	81.023	80.667	**84.368**	82.736
hypothyroid	93.506	93.638	94.884	**96.581**	95.520	95.573	96.077	**97.005**	95.838	96.077	96.288	**97.190**	96.368	96.368	96.527	**97.005**
ionosphere	80.071	80.087	**80.651**	77.500	79.206	76.921	80.063	**82.048**	79.492	**81.770**	79.754	81.484	80.611	80.627	84.056	**86.595**
iris	88.000	88.000	84.667	**90.667**	90.000	90.667	**94.667**	92.667	94.000	**94.667**	94.000	**94.667**	94.667	95.333	94.667	**96.667**
kr-vs-kp	92.991	92.679	93.742	**96.059**	95.089	95.182	96.966	**97.466**	97.060	97.029	97.185	**97.999**	96.904	96.872	97.529	**97.936**
labor	**73.000**	**73.000**	**73.000**	**73.000**	72.000	82.000	82.333	**85.667**	82.333	87.667	78.333	**91.000**	78.333	82.333	86.000	**93.333**
letter	74.480	74.410	**75.810**	73.130	76.080	76.055	**76.710**	75.050	76.710	76.630	**77.085**	75.930	77.020	77.025	**77.250**	77.100
lymphography	69.762	69.000	73.000	**75.000**	75.667	77.619	70.333	**80.286**	72.381	**74.333**	70.905	72.286	70.190	**76.381**	73.048	72.952
mushroom	99.606	99.618	99.606	**99.938**	99.926	99.902	99.963	**99.975**	99.963	99.963	99.975	**100.000**	**99.975**	**99.975**	**99.975**	**99.975**
optdigits	86.904	**92.295**	91.993	90.338	93.790	**93.897**	89.680	93.043	89.609	93.060	92.242	**94.093**	91.619	92.064	92.918	**94.075**
page-blocks	95.249	95.176	95.267	**96.218**	95.651	95.688	96.072	**96.400**	96.090	96.145	96.145	**96.382**	96.327	96.273	96.382	**96.602**
pendigits	90.384	90.293	92.476	**93.832**	93.932	93.941	94.805	**95.588**	94.814	94.787	95.297	**95.633**	95.051	95.042	95.579	**95.615**
pima_diabetes	74.745	74.226	**75.398**	75.137	**76.700**	**76.700**	76.569	76.179	76.958	76.567	76.048	**77.220**	75.530	76.699	76.828	**77.216**
postoperative	**62.222**	**62.222**	**62.222**	**62.222**	52.222	52.222	47.778	**54.444**	48.889	50.000	**56.667**	54.444	54.444	53.333	53.333	**57.778**
primary-tumor	30.089	28.583	29.153	**31.230**	**32.103**	30.053	31.533	28.012	31.533	**33.021**	30.089	32.727	33.342	33.057	**35.695**	**35.695**
segment	87.922	86.147	**91.082**	90.346	92.511	92.597	93.290	**94.113**	93.550	93.636	94.632	**95.022**	94.589	94.459	94.805	**95.325**
sick	94.406	94.273	90.041	**96.924**	96.023	96.023	96.235	**97.110**	96.262	96.262	90.368	**97.057**	96.288	96.315	90.686	**96.871**
solar-flare	63.528	64.014	**66.704**	66.662	67.297	67.360	65.812	**68.216**	65.411	66.011	**69.354**	67.680	68.476	68.382	**70.866**	69.583
sonar	**64.976**	61.548	63.071	59.762	**68.405**	62.595	66.857	67.333	69.714	67.762	71.190	**73.476**	67.310	64.452	70.810	**72.595**
soybean	73.325	75.539	80.211	**82.999**	82.726	84.192	85.507	**88.142**	86.974	86.969	86.984	**90.043**	87.415	86.087	87.709	**89.606**
spambase	88.024	88.502	89.741	**90.915**	90.480	90.567	91.284	**92.414**	91.284	91.241	92.067	**92.914**	91.849	91.849	92.306	**92.914**
spect	58.869	57.010	**61.455**	59.225	65.899	60.276	64.503	**67.675**	65.684	66.013	**71.554**	68.723	**70.242**	65.064	66.184	69.988
sponge	**74.286**	**74.286**	**74.286**	**74.286**	91.250	**92.500**	87.500	**92.500**	**92.500**	**92.500**	91.250	92.321	**93.750**	91.250	90.893	**93.750**
tae	33.125	33.125	**45.583**	35.042	42.417	42.375	43.083	**45.000**	46.375	44.333	50.292	**52.375**	47.625	47.625	48.875	**51.542**
tic-tac-toe	95.615	95.196	93.739	**97.599**	95.724	95.411	96.451	**97.808**	96.764	96.659	97.601	**98.121**	97.077	97.497	97.079	**98.329**
vehicle	70.931	70.461	**73.416**	71.765	76.366	76.015	78.146	**78.847**	77.434	77.319	78.266	**79.088**	78.382	77.555	78.615	**79.440**
vote	92.410	**93.795**	92.616	93.552	93.108	92.421	93.568	**96.089**	93.578	93.118	92.907	**96.094**	91.971	93.340	**94.017**	93.562
vowel	39.091	38.283	46.465	**47.980**	56.768	57.879	**65.455**	60.505	64.949	62.020	68.687	**68.990**	67.677	65.960	**73.333**	72.525
waveform	82.080	82.080	83.720	**84.260**	84.680	84.660	85.600	**86.140**	85.600	85.500	85.960	**86.480**	86.280	86.200	86.420	**86.740**
wine	92.712	93.791	89.902	**96.111**	92.712	94.379	95.523	**96.634**	94.935	94.967	**97.222**	97.190	96.667	**97.222**	96.634	96.078
wisconsin-breast	94.563	**94.990**	94.277	94.563	94.414	94.414	95.561	**96.565**	95.561	95.561	95.994	**96.565**	95.706	95.563	96.137	**96.708**
zoo	**79.273**	**79.273**	**79.273**	**79.273**	86.273	85.273	85.273	**94.091**	88.273	87.273	85.273	**93.091**	86.273	86.273	89.182	**94.091**
**Average**	71.096	71.221	72.768	**73.469**	74.925	75.248	76.528	**77.460**	77.085	76.916	78.155	**78.798**	77.785	77.422	79.345	**79.987**

Table S3. Classification accuracies of Logistic Model Trees (LMT) on four different *Ratios*.

Method / Dataset	***R*** **= 10%**				***R*** **= 20%**				***R*** **= 30%**				***R*** **= 40%**			
	Supervised	Semi-Supervised	Active Random	Combination	Supervised	Semi-Supervised	Active Random	Combination	Supervised	Semi-Supervised	Active Random	Combination	Supervised	Semi-Supervised	Active Random	Combination
anneal	83.287	83.745	84.071	**87.976**	85.629	89.086	89.642	**92.090**	89.532	93.773	91.427	**94.876**	90.200	93.657	90.870	**95.432**
arrhythmia	56.217	54.657	59.768	**64.184**	63.947	65.942	65.053	**66.604**	63.942	68.343	68.135	**69.242**	67.473	69.473	70.353	**71.488**
audiology	43.004	43.478	**54.862**	50.138	58.458	57.905	68.221	**69.881**	67.767	69.960	73.518	**76.542**	72.569	73.439	80.573	**81.324**
autos	44.333	41.810	**49.690**	48.238	56.048	55.595	58.881	**60.881**	61.857	**65.714**	61.833	65.167	64.810	63.786	69.714	**71.619**
balance-scale	86.866	86.073	**88.464**	86.861	**88.633**	87.837	85.763	87.688	86.557	88.162	88.003	**90.553**	88.477	87.683	**89.281**	88.971
breast-cancer	65.702	64.717	66.059	**66.453**	67.869	70.677	**71.675**	70.579	71.675	**73.067**	70.320	70.579	70.296	73.436	70.246	**76.576**
bridges-version1	**46.545**	**46.545**	**46.545**	**46.545**	56.000	58.000	**59.182**	55.091	64.000	62.909	63.000	**66.636**	61.000	63.091	**68.636**	64.818
bridges-version2	**44.545**	**44.545**	**44.545**	**44.545**	49.182	49.273	**61.818**	50.364	61.818	58.000	**62.000**	60.909	61.000	65.000	**68.545**	63.000
clevalend	75.194	73.161	**75.226**	74.849	77.871	78.570	**82.462**	78.849	82.473	80.828	**82.796**	82.161	80.505	80.538	**84.151**	82.172
cmc	45.349	45.621	**47.925**	47.588	47.794	46.705	**52.205**	50.442	51.864	49.421	**52.141**	51.663	**52.003**	51.257	51.871	51.535
column_2C	81.290	**81.613**	**81.613**	78.710	80.000	82.581	82.903	**84.194**	82.581	83.548	83.226	**85.484**	84.194	83.226	84.194	**86.129**
column_3C	82.581	80.000	81.613	**82.903**	83.871	82.581	83.226	**85.484**	**85.161**	82.258	84.516	84.194	85.161	85.484	84.516	**86.129**
credit-rating	82.464	**83.913**	83.623	83.623	84.203	**84.348**	84.203	84.203	84.348	**85.072**	84.058	84.638	83.333	84.638	84.348	**84.928**
cylinder-bands	62.222	**63.889**	58.333	62.407	61.667	62.037	**65.926**	63.519	65.370	68.148	69.444	**71.296**	68.333	70.926	72.963	**75.370**
dermatology	84.662	84.144	92.350	**95.661**	93.176	93.191	94.820	**97.553**	95.368	94.272	94.827	**97.560**	95.383	94.550	95.938	**97.275**
ecoli	73.556	75.597	75.936	**80.954**	81.569	80.963	83.930	**85.392**	83.930	84.207	**85.704**	**85.704**	85.410	86.301	86.578	**87.496**
flags	52.132	**53.053**	52.632	52.158	54.316	57.421	57.395	**61.447**	56.737	58.842	57.895	**59.868**	58.868	57.763	60.789	**63.500**
german_credit	68.200	68.800	70.300	**72.100**	69.900	72.500	**74.300**	73.500	**74.300**	73.700	73.500	73.800	73.600	73.000	**75.500**	74.100
glass	51.450	52.792	51.104	**53.355**	55.260	56.147	60.736	**60.779**	58.009	61.645	59.827	**62.597**	62.641	64.372	**66.320**	64.913
haberman	70.290	70.290	73.204	**73.839**	**74.828**	73.849	73.183	74.473	73.516	71.258	**73.860**	73.527	72.527	72.860	**74.151**	72.871
heart-statlog	76.667	75.556	75.556	**78.148**	77.037	75.185	79.630	**80.741**	79.630	75.926	80.370	**80.741**	77.778	79.259	81.852	**82.593**
hepatitis	76.167	78.167	79.417	**80.625**	80.083	76.833	80.000	**82.500**	79.917	80.583	78.708	**81.167**	80.542	79.250	81.292	**82.542**
horse-colic	77.695	78.799	**80.976**	80.165	82.883	**83.416**	78.791	81.779	79.602	**83.416**	82.598	80.968	81.216	**83.956**	83.393	83.408
hungarian-heart	80.299	81.000	80.690	**83.402**	80.011	**82.057**	78.609	82.046	79.966	78.276	**82.713**	79.000	81.379	80.322	81.667	**81.747**
hypothyroid	96.846	97.430	98.145	**99.099**	98.277	98.330	98.834	**99.125**	98.781	98.648	99.099	**99.125**	99.046	98.887	99.072	**99.549**
ionosphere	78.659	79.802	**83.492**	82.659	84.619	83.770	87.468	**88.611**	86.905	85.754	88.048	**90.040**	87.468	86.603	89.746	**92.595**
iris	88.000	80.000	88.000	**94.000**	93.333	92.000	**94.667**	93.333	95.333	94.667	**96.000**	**96.000**	96.000	**97.333**	96.667	94.667
kr-vs-kp	95.213	94.931	97.309	**98.687**	97.560	98.029	98.529	**99.531**	98.623	98.905	99.343	**99.750**	99.061	99.186	99.281	**99.750**
labor	**45.667**	**45.667**	**45.667**	**45.667**	68.333	57.667	**77.333**	71.000	77.333	76.333	69.667	**77.667**	69.667	75.000	79.000	**86.333**
letter	78.710	78.205	81.540	**83.150**	83.805	83.570	86.970	**88.650**	86.970	86.260	88.685	**90.500**	88.045	88.500	90.305	**91.415**
lymphography	65.476	65.619	**67.524**	66.333	70.762	70.190	72.810	**72.857**	76.286	76.333	**81.667**	78.190	76.952	79.619	80.333	**83.048**
mushroom	99.581	99.569	99.618	**99.815**	99.803	99.791	99.902	**99.938**	99.889	99.889	99.938	**99.963**	99.938	99.938	99.865	**99.975**
optdigits	93.879	93.630	94.698	**96.103**	95.231	95.391	95.854	**97.046**	95.819	95.872	96.637	**97.082**	96.370	96.637	96.779	**97.278**
page-blocks	95.194	95.669	96.072	**96.602**	95.815	96.072	96.382	**96.876**	96.181	95.998	96.602	**97.131**	96.620	96.565	96.730	**96.876**
pendigits	95.897	96.206	96.780	**97.853**	97.162	97.007	97.553	**98.317**	97.617	97.535	97.853	**98.426**	97.689	98.126	98.244	**98.372**
pima_diabetes	75.135	74.354	73.568	**76.827**	75.531	76.042	75.783	**77.473**	75.654	76.567	74.357	**77.086**	75.138	**77.348**	77.215	76.693
postoperative	**66.667**	**66.667**	**66.667**	**66.667**	68.889	66.667	**71.111**	70.000	70.000	67.778	64.444	**71.111**	66.667	65.556	66.667	**68.889**
primary-tumor	32.139	29.483	**37.442**	34.510	35.062	38.886	**41.542**	39.483	41.542	41.872	40.098	**42.772**	41.533	40.704	44.528	**46.889**
segment	92.641	92.857	**93.723**	93.463	93.723	93.117	93.853	**95.368**	94.545	94.502	94.762	**96.234**	94.892	95.065	96.364	**96.494**
sick	97.056	96.924	97.031	**98.568**	97.587	97.932	98.144	**98.860**	97.746	98.197	98.356	**98.966**	98.382	98.436	98.781	**99.019**
solar-flare	69.475	68.015	**70.649**	69.792	70.766	**70.923**	70.494	70.619	70.555	69.335	**72.418**	70.544	69.827	70.759	71.699	**72.902**
sonar	60.524	59.143	59.595	**60.571**	66.000	68.333	**73.024**	69.286	70.190	**74.524**	70.286	73.048	**76.476**	73.619	75.524	**76.476**
soybean	72.023	71.164	82.864	**83.148**	84.763	84.908	87.396	**90.034**	88.129	88.431	90.328	**92.965**	90.627	90.622	**92.969**	92.526
spambase	90.198	89.524	91.175	**92.176**	91.132	91.241	91.784	**92.610**	91.588	91.914	92.479	**92.849**	91.827	92.458	92.088	**93.067**
spect	61.932	61.747	60.792	**63.228**	68.337	68.784	**69.802**	68.584	71.839	73.029	74.548	**75.474**	72.974	71.717	**79.108**	76.902
sponge	**92.500**	**92.500**	**92.500**	**92.500**	**92.500**	**92.500**	**92.500**	**92.500**	**92.500**	**92.500**	**92.500**	**92.500**	**92.500**	**92.500**	**92.500**	**92.500**
tae	38.458	41.792	**47.042**	41.125	38.375	44.250	**47.708**	47.583	47.583	46.292	49.625	**55.000**	47.000	42.375	48.917	**51.583**
tic-tac-toe	75.569	71.607	**88.620**	88.297	94.047	86.846	93.934	**98.329**	93.524	92.277	97.285	**98.329**	96.762	94.779	97.495	**98.329**
vehicle	68.322	69.035	70.097	**72.584**	74.951	73.527	76.486	**77.433**	79.564	75.542	80.513	**80.739**	79.448	78.272	**80.405**	80.280
vote	**95.640**	**95.640**	95.407	**95.640**	94.958	94.498	95.412	**96.099**	95.412	95.407	**96.327**	95.640	**96.327**	95.180	95.862	95.634
vowel	48.384	46.162	**58.889**	55.556	67.273	67.071	75.960	**79.394**	76.566	75.354	83.636	**86.667**	82.424	82.424	**90.404**	88.182
waveform	84.820	84.840	84.940	**86.420**	85.660	86.040	86.180	**87.120**	86.180	85.960	86.320	**86.720**	86.160	86.560	86.620	**86.740**
wine	85.425	88.693	89.902	**93.235**	90.458	93.791	96.078	**96.634**	96.634	96.667	95.000	**98.889**	95.490	96.078	94.379	**96.667**
wisconsin-breast	93.565	92.565	94.992	**95.422**	95.990	95.282	95.418	**96.565**	95.275	95.422	**96.280**	95.994	95.992	95.994	95.565	**96.137**
zoo	**61.545**	**61.545**	**61.545**	**61.545**	74.273	73.273	79.273	**84.273**	85.273	84.273	86.273	**90.182**	87.273	84.273	89.273	**92.182**
**Average**	72.834	72.599	74.923	**75.576**	77.367	77.426	79.832	**80.247**	80.181	80.243	80.978	**82.263**	80.787	81.061	82.730	**83.416**

Table S4. Classification accuracies of LogitBoost on four different *Ratios*.

Dataset \ Method	*R* = 10%				*R* = 20%				*R* = 30%				*R* = 40%			
	Supervised	Semi-Supervised	Active Random	Combination	Supervised	Semi-Supervised	Active Random	Combination	Supervised	Semi-Supervised	Active Random	Combination	Supervised	Semi-Supervised	Active Random	Combination
anneal	93.869	93.874	94.878	**97.880**	96.213	95.433	97.548	**98.216**	97.548	97.770	98.327	**98.663**	97.994	97.658	98.105	**98.772**
arrhythmia	60.193	60.845	61.763	**64.841**	63.942	64.614	65.478	**68.826**	66.367	67.478	68.812	**70.812**	69.686	70.580	67.271	**73.686**
audiology	44.783	46.996	54.447	**58.024**	57.154	58.893	**69.960**	69.071	69.960	68.636	**73.498**	73.439	73.893	74.763	79.684	**80.968**
autos	43.881	42.429	48.310	**51.143**	54.976	54.952	59.976	**62.905**	60.476	61.905	68.667	**70.762**	68.238	66.286	72.548	**74.500**
balance-scale	79.841	79.672	81.429	**86.400**	83.671	84.165	84.501	**87.693**	86.249	84.324	86.073	**86.897**	87.207	85.591	**87.673**	87.522
breast-cancer	**65.382**	65.025	64.310	65.049	**71.330**	66.392	68.498	69.581	67.796	70.567	**70.985**	70.936	72.044	70.985	**72.734**	71.330
bridges-version1	**44.545**	**44.545**	**44.545**	**44.545**	51.364	54.273	**60.273**	59.091	57.182	62.818	62.727	**64.909**	**64.727**	60.727	63.455	61.909
bridges-version2	**45.636**	**45.636**	**45.636**	**45.636**	42.727	44.636	**56.273**	51.091	61.727	59.818	59.182	**63.909**	60.182	62.000	**67.000**	62.000
clevalend	74.527	71.849	73.151	**76.882**	77.559	76.882	**81.172**	77.161	**80.839**	77.161	79.495	79.462	78.172	78.183	76.516	**79.194**
cmc	49.356	50.780	52.751	**52.956**	52.683	51.800	**55.666**	53.628	**55.462**	52.679	54.244	54.713	**55.058**	54.039	54.106	54.239
column_2C	78.065	77.419	75.161	**79.032**	79.355	78.387	80.000	**80.968**	78.387	81.290	**81.935**	81.613	81.613	79.677	**83.548**	80.968
column_3C	76.129	76.452	75.806	**80.645**	**81.290**	80.000	**81.290**	80.968	80.323	80.645	78.387	**81.935**	81.613	81.290	81.290	**82.258**
credit-rating	81.739	81.304	**83.623**	82.899	84.493	85.507	85.507	**85.652**	84.928	84.638	85.217	**85.797**	83.623	84.203	**86.377**	85.507
cylinder-bands	62.593	**67.407**	**67.407**	**67.407**	66.481	66.481	69.444	**70.370**	**69.630**	68.704	69.444	69.259	69.444	69.815	**72.778**	69.815
dermatology	89.902	89.069	93.161	**93.709**	95.105	94.820	94.827	**96.194**	94.827	96.186	94.827	**96.456**	95.375	95.916	**96.471**	**96.471**
ecoli	67.638	66.417	71.791	**75.036**	75.303	74.421	79.153	**83.922**	79.153	80.089	81.542	**83.610**	82.745	81.524	82.139	**83.930**
flags	49.079	49.079	52.658	**52.763**	49.632	52.763	54.184	**56.895**	57.368	55.711	58.868	**64.026**	58.342	57.868	59.842	**60.395**
german_credit	70.300	69.900	69.600	**71.800**	69.700	69.000	**72.700**	72.600	**72.700**	71.700	71.300	71.800	72.100	**72.200**	68.800	71.900
glass	51.926	51.948	54.329	**55.260**	60.303	61.753	63.636	**64.935**	61.732	65.022	63.095	**67.359**	60.281	65.043	69.567	**70.130**
haberman	68.269	65.978	69.882	**70.237**	72.194	70.226	**72.204**	71.860	72.882	71.237	73.538	**75.505**	71.903	74.495	73.204	**74.817**
heart-statlog	70.000	72.222	71.852	**75.926**	74.444	75.556	79.630	**80.370**	78.519	75.926	76.667	**82.963**	77.407	76.667	77.037	**80.741**
hepatitis	77.292	78.542	**79.292**	74.167	75.333	**79.958**	79.333	78.042	79.875	80.625	76.583	**81.958**	77.250	78.458	**87.000**	84.458
horse-colic	70.661	70.646	74.459	**76.044**	76.336	77.973	76.907	**81.239**	74.167	74.752	77.432	**81.261**	78.566	78.814	77.733	**83.138**
hungarian-heart	75.218	78.977	**79.322**	79.230	78.609	**79.287**	74.839	78.276	74.897	76.885	79.207	**79.989**	78.230	80.655	79.644	**80.989**
hypothyroid	98.065	97.986	98.542	**99.602**	98.913	98.940	99.205	**99.576**	99.178	99.099	99.311	**99.549**	99.231	99.205	99.390	**99.496**
ionosphere	76.659	76.659	84.357	**85.492**	86.040	85.762	89.167	**92.603**	88.913	87.468	**91.183**	90.881	89.190	89.468	**92.032**	89.762
iris	89.333	90.667	89.333	**94.667**	94.000	**95.333**	**95.333**	94.000	96.000	96.000	94.667	**96.667**	**96.000**	95.333	95.333	95.333
kr-vs-kp	93.554	93.712	93.836	**94.055**	93.774	93.805	93.836	**94.306**	93.836	**93.930**	93.836	93.836	**93.836**	93.680	**93.836**	**93.836**
labor	**65.333**	**65.333**	**65.333**	**65.333**	67.667	76.000	73.667	**81.333**	73.667	73.667	78.333	**87.667**	78.333	83.667	84.000	**86.000**
letter	70.955	69.870	**71.900**	68.925	72.175	70.970	**72.845**	70.505	72.845	71.450	**73.100**	71.920	72.940	71.960	**73.045**	72.315
lymphography	69.571	69.571	70.333	**75.762**	76.905	**77.048**	76.238	75.619	**81.762**	77.571	80.905	77.619	79.619	78.381	**80.429**	80.333
mushroom	98.560	98.621	98.621	**99.680**	99.003	98.375	98.979	**99.557**	98.979	98.523	98.905	**99.508**	98.659	98.178	98.412	**99.015**
optdigits	88.754	89.039	90.160	**90.801**	90.142	90.552	91.228	**91.957**	91.281	91.157	91.388	**92.082**	91.174	91.174	91.495	**92.082**
page-blocks	95.669	95.359	96.017	**96.949**	96.364	96.236	96.455	**97.205**	96.437	96.071	96.455	**97.058**	96.528	96.638	96.711	**96.985**
pendigits	90.011	89.929	90.784	**90.866**	91.203	90.366	91.430	**91.921**	91.203	90.648	**92.313**	92.140	91.985	90.994	92.031	**92.376**
pima_diabetes	71.488	72.531	71.615	**73.184**	75.005	75.530	**76.567**	73.060	**75.660**	74.352	73.698	74.484	**74.875**	74.612	74.609	73.698
postoperative	**60.000**	**60.000**	**60.000**	**60.000**	**61.111**	56.667	58.889	55.556	**62.222**	56.667	57.778	**62.222**	**61.111**	56.667	60.000	**61.111**
primary-tumor	33.904	35.089	**40.080**	37.184	39.189	39.474	42.451	**42.763**	42.451	44.813	43.307	**45.107**	43.039	44.207	**46.916**	44.251
segment	92.251	92.338	93.377	**94.545**	93.810	93.074	94.286	**96.190**	94.589	93.593	95.022	**95.671**	95.065	95.065	95.238	**95.498**
sick	97.587	97.693	97.375	**98.011**	97.879	97.693	97.879	**98.038**	97.879	**98.091**	97.906	97.852	**97.905**	97.852	97.879	**97.905**
solar-flare	67.981	69.039	**71.162**	70.742	70.771	69.730	70.566	**71.409**	70.925	69.966	71.491	**72.098**	72.108	71.146	**73.091**	72.019
sonar	61.952	62.905	61.929	**65.405**	**67.738**	65.357	66.095	67.286	73.071	72.095	74.976	**75.952**	72.095	74.071	**77.476**	77.357
soybean	72.621	73.050	**84.471**	84.041	86.377	86.522	89.011	**91.647**	89.450	89.889	91.799	**93.700**	90.774	91.650	92.679	**93.114**
spambase	90.132	89.632	90.936	**91.849**	90.849	90.524	91.219	**91.589**	91.262	90.719	**91.806**	91.741	91.415	91.219	91.675	**92.023**
spect	66.108	65.175	**68.536**	66.108	67.837	68.577	67.171	**75.166**	70.791	68.531	**73.654**	73.020	72.534	74.085	**76.553**	73.097
sponge	**88.214**	**88.214**	**88.214**	**88.214**	**92.500**	**92.500**	**92.500**	**92.500**	**93.750**	**93.750**	92.500	**93.750**	**93.750**	92.500	**93.750**	**93.750**
tae	32.417	33.750	**39.667**	37.042	40.333	39.708	43.667	**44.333**	42.375	41.083	**45.667**	44.333	43.708	46.958	**47.625**	46.917
tic-tac-toe	70.461	69.942	71.708	**71.925**	72.544	70.765	73.596	**73.805**	**74.534**	72.963	73.693	74.011	73.069	72.753	72.751	**74.012**
vehicle	61.473	59.817	65.486	**65.612**	65.616	65.496	68.209	**69.508**	67.499	67.501	**71.289**	69.272	**70.342**	70.220	69.986	70.209
vote	94.265	93.573	94.027	**96.094**	94.514	94.503	95.872	**95.883**	95.407	95.867	94.271	**96.105**	94.725	94.952	95.862	**96.332**
vowel	45.051	48.485	53.030	**53.131**	58.687	57.273	**64.343**	62.121	63.030	61.717	65.354	**66.162**	66.263	64.040	69.192	**69.293**
waveform	81.020	80.140	81.380	**82.180**	82.020	81.460	82.200	**82.460**	82.200	81.920	82.660	**83.020**	82.360	82.740	82.180	**83.140**
wine	86.536	85.425	83.758	**88.301**	91.569	93.758	96.111	**98.333**	95.556	95.523	96.111	**97.190**	96.667	96.111	96.111	**97.745**
wisconsin-breast	94.420	94.275	93.563	**94.994**	94.849	95.135	95.277	**95.422**	95.277	94.563	95.280	**95.708**	94.851	95.280	**95.996**	95.994
zoo	**76.273**	**76.273**	**76.273**	**76.273**	77.273	78.273	80.273	**86.182**	81.273	81.273	87.182	**94.091**	87.182	86.273	88.273	**94.182**
**Average**	72.208	72.384	74.098	**75.172**	75.943	76.065	77.956	**78.752**	78.296	77.946	79.198	**80.590**	79.219	79.246	80.529	**80.888**

© 2019 by the authors. Submitted for possible open access publication under the terms and conditions of the Creative Commons Attribution (CC BY) license (http://creativecommons.org/licenses/by/4.0/).
